# The *Drosophila* surface glia transcriptome: evolutionary conserved blood-brain barrier processes

**DOI:** 10.3389/fnins.2014.00346

**Published:** 2014-11-07

**Authors:** Michael K. DeSalvo, Samantha J. Hindle, Zeid M. Rusan, Souvinh Orng, Mark Eddison, Kyle Halliwill, Roland J. Bainton

**Affiliations:** ^1^Department of Anesthesia and Perioperative Care, University of California San FranciscoSan Francisco, CA, USA; ^2^Janelia Farm Research Campus, The Howard Hughes Medical InstituteAshburn, VA, USA; ^3^Pharmaceutical Sciences and Pharmacogenomics, University of California San FranciscoSan Francisco, CA, USA

**Keywords:** blood-brain barrier, transcriptome, surface glia, Affymetrix array

## Abstract

Central nervous system (CNS) function is dependent on the stringent regulation of metabolites, drugs, cells, and pathogens exposed to the CNS space. Cellular blood-brain barrier (BBB) structures are highly specific checkpoints governing entry and exit of all small molecules to and from the brain interstitial space, but the precise mechanisms that regulate the BBB are not well understood. In addition, the BBB has long been a challenging obstacle to the pharmacologic treatment of CNS diseases; thus model systems that can parse the functions of the BBB are highly desirable. In this study, we sought to define the transcriptome of the adult *Drosophila melanogaster* BBB by isolating the BBB surface glia with fluorescence activated cell sorting (FACS) and profiling their gene expression with microarrays. By comparing the transcriptome of these surface glia to that of all brain glia, brain neurons, and whole brains, we present a catalog of transcripts that are selectively enriched at the *Drosophila* BBB. We found that the fly surface glia show high expression of many ATP-binding cassette (ABC) and solute carrier (SLC) transporters, cell adhesion molecules, metabolic enzymes, signaling molecules, and components of xenobiotic metabolism pathways. Using gene sequence-based alignments, we compare the *Drosophila* and Murine BBB transcriptomes and discover many shared chemoprotective and small molecule control pathways, thus affirming the relevance of invertebrate models for studying evolutionary conserved BBB properties. The *Drosophila* BBB transcriptome is valuable to vertebrate and insect biologists alike as a resource for studying proteins underlying diffusion barrier development and maintenance, glial biology, and regulation of drug transport at tissue barriers.

## Introduction

Endothelial cells constituting the capillaries of the vertebrate central nervous system (CNS) have special properties that enable a potent blood-brain barrier (BBB). The BBB preserves CNS homeostasis by preventing the entry of harmful molecules and facilitating the passage of essential molecules such as metabolites. While the brain vascular endothelial cells are the anatomic BBB, all members of the neurovascular unit (NVU—endothelial cells, pericytes, astrocytes, extracellular matrix, and neurons) are thought to contribute to the development and maintenance of BBB processes (Janzer and Raff, 1987; Sobue et al., 1999; Armulik et al., 2010; Daneman et al., 2010b). That being said, the barrier functions of the BBB are largely provided by vascular endothelial machinery: intercellular protein complexes, active efflux transporters, and carrier-mediated transporters (Zlokovic, 2008). In particular, the formation of tight junction (TJ) complexes between endothelial cells renders paracellular fluid flux impossible (Hirase et al., 1997; Saitou et al., 2000; Nitta et al., 2003). Also, endothelial cell expression of ABCB1 (i.e., MDR1/P-glycoprotein), an ATP-binding cassette (ABC) transporter found at the luminal surface, is responsible for the efflux of unwanted substrates back into the blood (Cordon-Cardo et al., 1989; Loscher and Potschka, 2005), and expression of SLC2A1 (i.e., GLUT1), a solute carrier (SLC) transporter found at both surfaces, shuttles glucose between the blood and the brain (Boado and Pardridge, 1990; Pardridge et al., 1990). Characterizing the entire repertoire of genes underlying BBB physiology is of paramount importance given that (1) little is known about the regulatory mechanisms that grant the BBB its properties, (2) the etiologies of numerous CNS diseases that include BBB dysfunctions (Daneman, 2012), and (3) CNS disease treatments that depend on efficient delivery of therapeutics across the BBB (Pardridge, 2005).

Genomic approaches to characterizing cellular BBB structures have yielded important resources for understanding the BBB. Enerson and Drewes (Enerson and Drewes, 2006) isolated blood microvessels, which contained endothelial cells, pericytes, extracellular matrix, and remnant astrocytic end feet, from rat brains and used serial analysis of gene expression to produce the first profile of a vertebrate BBB transcriptome. Daneman et al. (2010a) used fluorescence activated cell sorting (FACS) to purify brain vascular endothelial cells from mice and used Affymetrix GeneChips to survey the transcriptome of the endothelial BBB component. Both of these studies identified known BBB transcripts, but more importantly, they also identified novel transcripts enriched at the BBB. Indeed, the BBB genes involved in CNS disease progression are not known in most cases (Daneman, 2012). In addition, strategies for delivering pharmaceuticals to the CNS have often centered on disrupting previously identified BBB drug efflux transporters such as ABCB1, but these direct strategies have been met with limited success (de Vries et al., 2007; Wu et al., 2011; Lin et al., 2013). Thus, in-depth exploration of the genes that regulate the integrated chemical protection physiologies present at the BBB is needed for overcoming BBB-related challenges.

**Figure 1:**
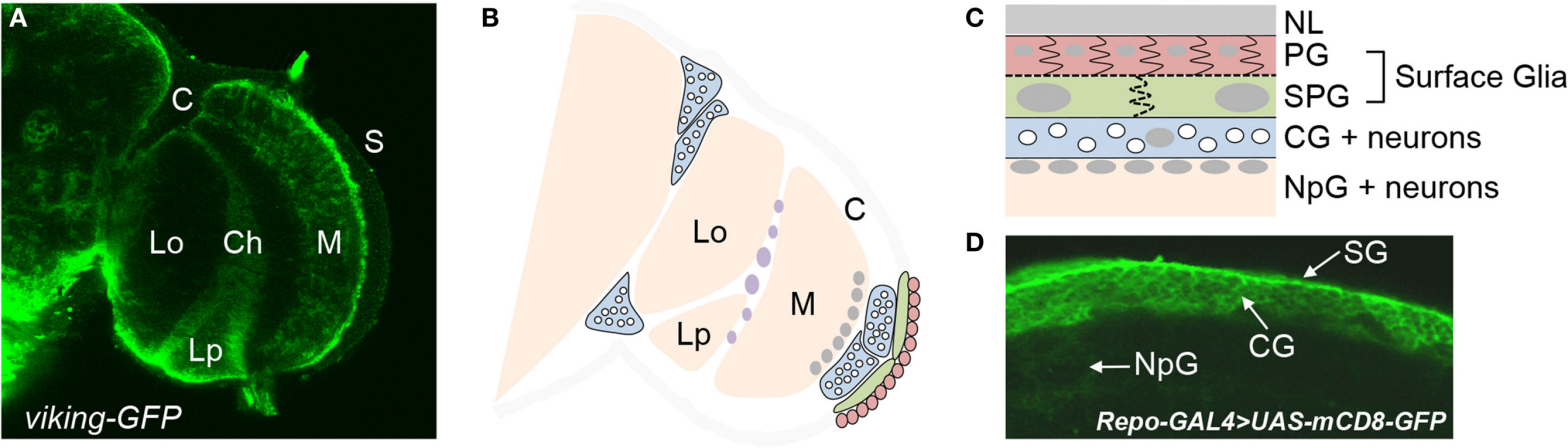
**Types of glia in the adult brain of *Drosophila.* (A)** Different regions of the optic lobe of an adult brain as visualized by a GFP-fusion protein of the pan-glial gene, *viking*. Glia are located at the brain surface (S) and the cortex (C), in addition to the medulla (M), lobula (Lo), and lobular plate (Lp) neuropils. The chiasm (Ch) is a glial region dividing the different neuropils. **(B)** A schematic of the optic lobe illustrating the different glial subtypes. Perineurial glia (PG) are seen in red, subperineurial glia (SPG) in green, and cortex glia (CG) in blue. The white circles within each cortex glia are neuronal cell bodies. Nuclei of neuropil glia (NpG) are in gray at the surface of the medulla neuropil. Nuclei of chiasm glia are in purple. **(C)** A cross section of the brain surface moving from the neural lamella (NL) to the neuropil. **(D)** Different glial subtypes at the brain surface as visualized by *UAS-mCD8-GFP* under control of the pan-glial driver *repo*-GAL4.

A major limitation to studying the hundreds of BBB genes in vertebrates is that obtaining mutants is both time consuming and costly. For this reason, we have focused on using the *Drosophila* BBB as a model for the study of BBB function. Previous studies have shown that the insect BBB is analogous to the vertebrate BBB (Stork et al., 2008; Mayer et al., 2009; DeSalvo et al., 2011). The *Drosophila* CNS is similarly protected by a BBB with one main noteworthy difference: insects have an open circulatory system where molecules are dissolved in a fluid called hemolymph that bathes organs, instead of being distributed in a vascular system. For this reason, the insect BBB encapsulates the CNS (Stork et al., 2008). Resembling the multiple cell type architecture of the vertebrate NVU, the insect BBB is composed of two glial subtypes collectively known as surface glia—the apical perineurial glia (PG) and basal subperineurial glia (SPG) (Stork et al., 2008) (Figure 1). In addition, endothelial cells of the vertebrate NVU are surrounded by an extracellular matrix in which pericytes are embedded, and in the insect glial BBB, a similar matrix termed the neural lamella is apical to the PG (Stork et al., 2008). Moreover, cellular junctions precluding small molecule diffusion between cells are paramount to BBB function. The insect equivalent of the vertebrate TJ is the septate junction (SJ), consisting of similar molecular components, present between the SPG to prevent paracellular molecule diffusion (Wu and Beitel, 2004). Finally, the ABC drug efflux transporter Mdr65 is expressed in the SPG and is involved in chemical protection of the CNS, analogous to the function of its vertebrate homolog ABCB1 (Mayer et al., 2009). Fundamental CNS homeostasis requirements imposed across animal phyla suggest that numerous conserved BBB physiologies will likely be discovered in *Drosophila*.

We present here a transcriptome of the *Drosophila* BBB surface glia. We assess the relative enrichment of genes expressed at the BBB by comparing the surface glia transcriptome to that of all brain glia, brain neurons, and whole brains. By way of example we show that many genes found in the highly purified transcriptome are both highly expressed and enriched in the surface glia. We use gene set enrichment analyses to validate our surface glia transcriptome and show that the *Drosophila* surface glia possess many cellular processes and molecular functions also resident at the vertebrate BBB. Furthermore, we demonstrate that our data can be used to find many novel genes expressed in the surface glia. Lastly, we use BLAST to identify genes expressed in both invertebrate and vertebrate BBBs, pointing to likely evolutionary conserved mechanisms of BBB function that can now be tested using the *Drosophila* model system. Together with the vast and readily available molecular genetic tools for *Drosophila*, our data provide a wealthy resource for rapidly screening through a large group of conserved BBB proteins for phenotypes of interest.

## Materials and methods

### Fly genetics

The following GAL4/UAS-GFP reporter lines were used to facilitate FACS of different CNS cell types: (1) *9-137*-GAL4 (a P-element insertion line found in a screen of a large P-GAL4 collection (Ulrike Heberlein, Janelia Farm Research Campus, VA) that drives expression in the surface glia), crossed to pJFRC2, a previously published *UAS-mCD8-GFP* line (Pfeiffer et al., 2010) available from the Bloomington Stock Center; (2) *repo*-GFP, a recombinant line of *repo*-GAL4 and *UAS-mCD8-GFP* that drives expression in all glia (Marc Freeman, UMass, MA); and (3) *elav*-GFP, a recombinant line of *elav*-GAL4 and *UAS-mCD8-GFP* that drives expression in all neurons. Whole brain controls were of wildtype Canton-S. For anatomic characterization of *9-137*-GAL4, we crossed this line to nuclear-localized GFP (*UAS-StingerGFP*) (Barolo et al., 2000) in addition to pJFRC2. FlyTrap lines are available from http://flytrap.med.yale.edu/ using genotype IDs stated in **Figure 5**.

### Whole brain imaging

Whole brain confocal images were acquired using previously reported methods (Mayer et al., 2009; DeSalvo et al., 2011; Pinsonneault et al., 2011). Briefly, flies were injected with 12.5 mg/ml 70 kDa Dextran Texas Red® (Invitrogen, D1864) and left to recover overnight. Dextran labeling of the brains allows for demarcation of the surface glia barrier. Fly heads were fixed *in situ* for 15 min with 3.7% paraformaldehyde prior to brain dissection. Fixed brains were then incubated for 1 h at room temperature in a blocking buffer (PBS containing 5% goat serum and 4% Tween® 20), and then probed with rabbit anti-GFP antibody (Abcam ab6556, 1:1000 dilution) overnight at 4°C. Brains were washed three times for 30 min in 1× PBS and incubated with FITC-conjugated goat anti-rabbit antibody (Jackson Immuno Research Laboratories, 1:100 dilution) for 45 min at room temperature. Brains were washed three times for 45 min in PBS and mounted in Dakocytomation Fluorescent Mounting Medium. Brains were visualized on a Zeiss LSM510 confocal microscope at 40× magnification.

### Tissue processing and FACS

Brains were dissected under Schneider's media (SM) containing 1% BSA (filter sterilized) and transferred directly to a tube containing ice-cold 500 μL SM/BSA. Each tube contained 10–15 brains. Brains were washed with 1 mL SM/BSA and re-suspended in 220 uL SM/BSA. Collagenase A (Roche No. 10103586001) and DNase I were added to final concentrations of 2 mg/mL and 20 units, respectively. Brains were dissociated at 37°C in a Thermomixer according to the following conditions: 1000 rpm for 20 min (*elav*/neurons), 500 rpm for 20 min (*repo*/all glia), and 500 rpm for 5 min (*9-137*/surface glia). EDTA (pH 7) was added to a final concentration of 5 mM to inactivate the collagenase. Dissociated tissue was filtered through 100 μm filter (70 μm for neurons) and immediately sorted using a 100 μm nozzle on a BD FACSAria at the Laboratory for Cell Analysis at UCSF. Except whilst in the Thermomixer and the FACS machine, brains/cells were kept on ice.

FACS sorting was performed according to the following gating procedure: (1) an initial SSC-A/FSC-A gate to minimize debris; (2) a FSC-W/FSC-A gate to minimize doublets and large cellular aggregates; and (3) a PE-A/FITC-A gate to choose only GFP-positive cells. GFP-positive cells from multiple tubes were sorted into a single tube containing a small volume of sheath fluid. These cells were sorted again to increase purity, and during this sort, the initial scatter gate was modified to capture neurons and glia according to their unique scatter properties (see **Figure 3**). At this step, cells were sorted directly into ice-cold RNA lysis buffer (Ambion RNAqueous Micro Kit). When possible, re-sorted cells were analyzed again to determine their final purity levels. Of the five replicates for *elav*, an average of 9705 cells were sorted at an average purity of 95%; *repo*, 8849 cells at 96% purity; and *9-137*, 6093 cells at 93% purity.

### Microarray data acquisition and analysis

Each replicate for the microarray analysis represented sorted cells originating from different growth bottles and processed on different days. Five total replicates were run for each of the following genotypes: wildtype whole brain, and sorted GFP-positive cells from *repo* (all glia), *elav* (neurons), and *9-137* (surface glia). RNA was isolated using Ambion RNAqueous Micro columns and amplified using NuGEN's Ovation FFPE WTA System. Amplified RNA was processed and hybridized to Affymetrix *Drosophila* Genome 2.0 GeneChips at the Gladstone Institutes Genomics Core facility. All microarray data analysis was performed using R/Bioconductor packages (Gentleman et al., 2004). CEL files were read and raw data normalized using RMA in the affy package (Irizarry et al., 2003; Gautier et al., 2004). Prior to statistical analysis, probes were filtered if the Present sum was less than 4 for all genotypes; in other words, a probe needed to be expressed in at least one genotype to be included. This metric was shown to significantly reduce false positives (McClintick and Edenberg, 2006). 7090 probes were filtered according to this criterion. The remaining probes were used for statistical analyses in the limma package (Smyth, 2004). Pairwise comparisons were performed for all possible combinations, and fold changes and standard errors were estimated by fitting a linear model for each gene. Empirical Bayes smoothing to the standard errors was applied and differentially expressed genes were chosen according to an FDR-adjusted *P* < 0.05. Lists of differentially expressed genes were trimmed to ensure that genes were indeed expressed in the enriched genotype (expression > 100 AND Present sum ≥ 4). Gene set enrichment analyses of the differentially expressed genes were performed using default settings in DAVID Bioinformatics (Huang et al., 2009a,b). Significantly enriched gene sets were identified using a Benjamini-adjusted *P* < 0.05.

### BLAST analysis

Using Ensembl Biomart (Kinsella et al., 2011), RefSeq protein IDs were retrieved for 144 known mouse BBB proteins according to Daneman (2012) and Zlokovic (2008). We chose to ignore proteins listed by Daneman (2012) that were up-regulated in the mouse BBB during disease. A fasta file containing all protein sequences was generated using Batch Entrez (http://www.ncbi.nlm.nih.gov/sites/batchentrez), and these sequences were compared to a BLAST-able database containing all *Drosophila* proteins using the BLAST+ command line (blastp with a E-value cutoff of 10^−5^). The blast2table perl script was used to parse the output file showing only the top HSP for each BLAST hit. We then linked the RefSeq protein ID for each fly BLAST hit to its corresponding Affymetrix probe IDs, which allowed us to annotate each BLAST hit with its expression and enrichment values in the surface glia transcriptome. Positive expression in the surface glia was assessed by having a Present sum ≥4 AND an expression level ≥100. Surface glia enrichment was assessed by having a positive enrichment relative to neurons OR whole brain.

## Results

### Isolation of surface glia RNA

**Figure 2:**
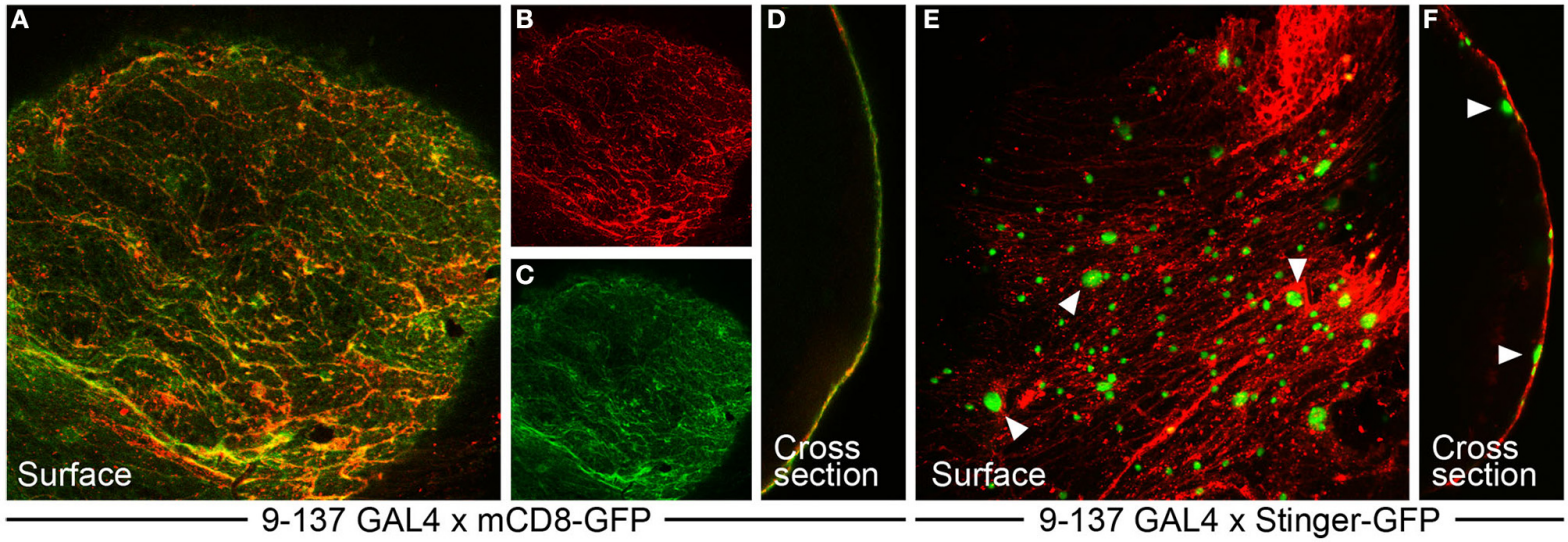
**The *9-137* P-GAL4 line specifically drives expression in the surface glia layer**. **(A–D)** *9-137*-GAL4 crossed to mCD8-GFP (membrane-bound) shows hallmarks of surface glia expression. **(A–C)** GFP expression at the surface colocalizes with dextran, which marks the boundaries of the PG cells in a characteristic flagstone pattern. **(D)** The cross-section image shows complete overlap between dextran and GFP. **(E,F)** *9-137*-GAL4 crossed to Stinger-GFP (nuclear-localized) demarcates small PG and large SPG nuclei. Arrowheads mark representative large SPG nuclei. **(F)** The cross-section image shows PG nuclei embedded in the dextran layer with SPG nuclei positioned below the dextran/PG layer.

**Figure 3:**
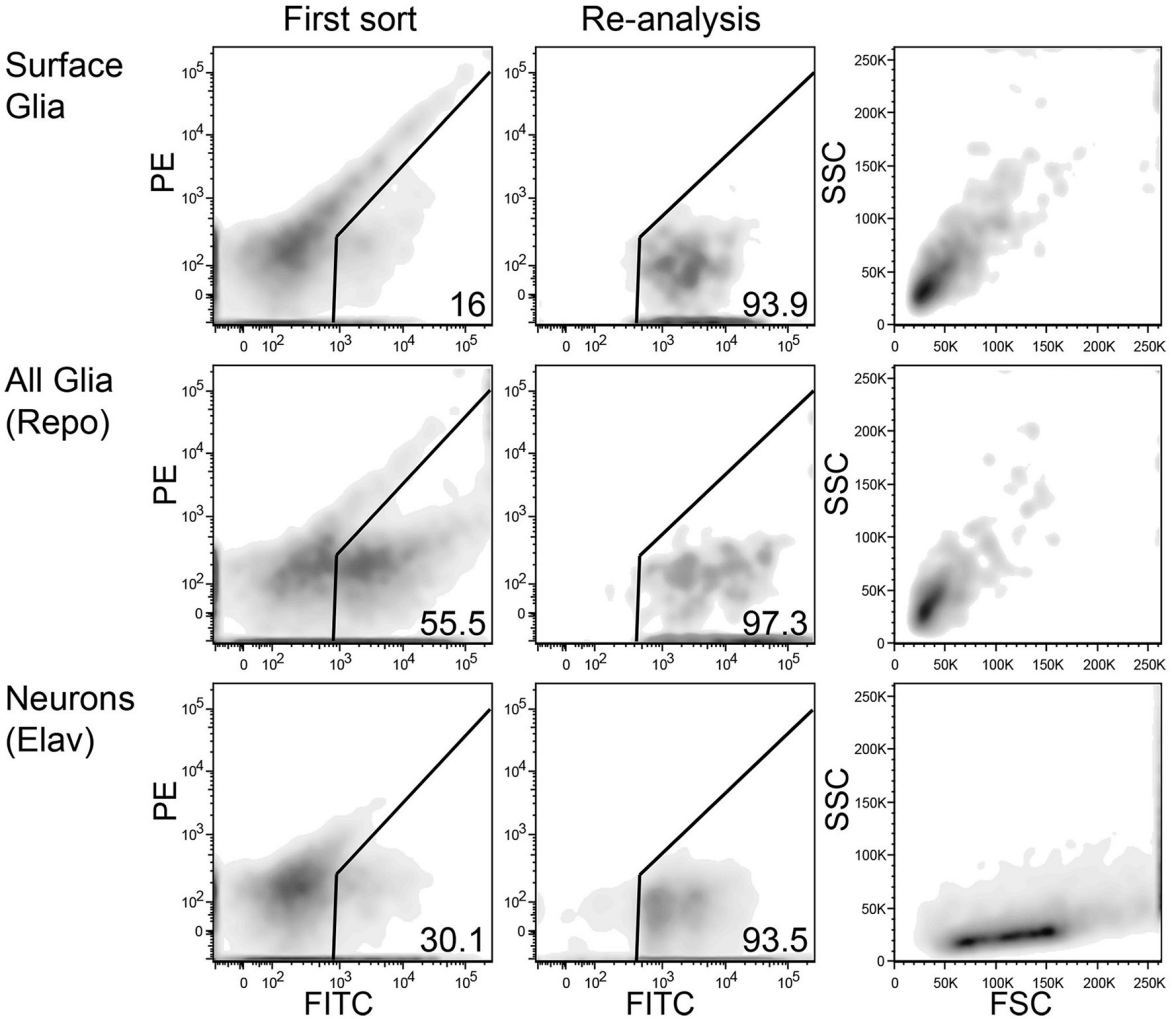
**Representative FACS density plots of sorted neurons and glia from adult *Drosophila* brains**. Left panels show red (PE) vs. green (FITC) fluorescence of dissociated brain cells. A population of autofluorescent particles extends diagonally across each plot. The GFP-positive gate lies to the right of the autofluorescence axis. The middle panels also show red vs. green fluorescence, but these plots demonstrate the high purity of sorted GFP-positive cells. The re-sorting of GFP-positive cells leads to high levels of purity. Right panels show side (SSC) and forward (FSC) scatter for the highly pure sorted cells in the middle panels. Interestingly, neurons (bottom) and glia (top and middle) have unique, nearly exclusive scatter properties.

To purify the BBB surface glia from adult *Drosophila* brains, we used a GAL4/UAS genetic approach (Brand and Perrimon, 1993) to fluorescently label the surface glia. To do this, we first identified the *9-137* enhancer trap line, which drives GAL4 expression specifically in the surface glia. When crossed to UAS-GFP reporter lines, the *9-137*-GAL4 results in specific GFP labeling of the PG and SPG (Figure 2), allowing the specific isolation of surface glia to a purity of >90% using FACS (Figure 3). We used a similar protocol to isolate neurons and a more inclusive population of glia. All brain glial subtypes (Figure 1), including surface glia, were isolated from flies expressing *UAS-mCD8-GFP* under the control of the pan-glial driver *repo*-GAL4 (Xiong et al., 1994); neurons were specifically isolated using the pan-neuronal driver *elav*-GAL4 (Campos et al., 1987; Robinow and White, 1988). Total RNA from five replicate samples for each of surface glia, all glia, neurons, and whole brains were amplified and hybridized to Affymetrix *Drosophila* Genome 2.0 GeneChips for downstream transcriptomic analysis. All microarray data is deposited in Gene Expression Omnibus (GSE45344), and the master matrix of filtered normalized data used as input for statistical analyses is found in Table S1. While we chose to focus on the surface glia-enriched transcriptome in this study, the comprehensive data set generated here is valuable for other avenues of research. For example, the data may provide broad insight into glial biology when analyzed for gene enrichment of additional glial subtypes such as the adult cortex and neuropil glia (Figure 1).

### The surface glia transcriptome

To gain insight into the genes required for specialized BBB functions in *Drosophila*, we first looked at transcript abundance in the surface glia. The microarray data were normalized using RMA in the R/Bioconductor package affy (Irizarry et al., 2003; Gautier et al., 2004). Table 1 reports the 50 most abundant transcripts in the surface glia together with their ratiometric enrichments relative to whole brain, neurons, and all glia. We acknowledge that Affymetrix expression signals are not perfect reflections of gene expression levels since signal values are the result of several factors both biological and technical. However, this list does appear to reveal specialized gene expression in the surface glia. For example, extracellular matrix collagens (*vkg* and *Cg25C*) are both highly expressed and highly enriched, and a SLC5 sodium-iodide symporter (*CG5687*) is the most enriched gene among the top 50 most abundant surface glia transcripts. Therefore, the data in Table 1 suggest that Affymetrix signal abundance for the surface glia samples is somewhat indicative of surface glia functional requirements.

**Table 1:** **The 50 highest expressed genes in the surface glia (SG) transcriptome**.

**Probe**	**Symbol**	**Signal**	**SG/B**	**SG/N**	**SG/AG**	**Function**
1639180_at	*Arc1*	17,218 ± 1568	1.52	10.67	9.74	Nucleic acid/zinc ion binding
1627489_a_at	*CG10433*	16,152 ± 1251	1.59	54.99	1.41	Defense response
1639614_s_at	*RpL41*	15,533 ± 1949	0.77	1.35	1.20	Cytosolic large ribosomal subunit
1639145_s_at	*Atpα*	15,084 ± 1190	1.10	1.17	0.99	Na,K ATPase alpha subunit
1633109_at	*CR42862*	14,265 ± 4275	1.04	1.06	1.07	Non-protein-coding gene
1639694_s_at	*Arc1*	13,937 ± 2103	1.64	35.22	25.58	Nucleic acid/zinc ion binding
1625719_at	*Atpalpha*	13,363 ± 1697	0.89	0.90	0.86	Na,K ATPase alpha subunit
1637526_s_at	*Gγ30A*	13,201 ± 2297	0.93	0.72	0.75	GPCR signaling
1639229_at	*vkg*	12,278 ± 3159	8.89	161.51	1.96	Collagen
1630150_s_at	*Cg25C*	12,239 ± 2512	6.78	396.13	2.28	Collagen
1636548_at	*blw*	11,894 ± 2063	1.10	1.21	0.90	Mitochondrial ATP synthase α-subunit
1629065_s_at	*Vha26*	11,814 ± 678	1.35	1.16	1.13	V-ATPase E subunit
1632873_at	*MtnA*	11,230 ± 3013	0.89	30.51	1.34	Metal ion binding
1632683_s_at	*copiaGIP*	11,090 ± 1461	1.36	1.82	1.32	Transposable element
1630946_at	*Vha16*	11,017 ± 694	0.90	1.16	1.09	V-ATPase C subunit
1632248_s_at	*CycG*	10,855 ± 795	1.48	1.11	1.14	Cell cycle
1630941_s_at	*sesB*	10,802 ± 1476	0.86	1.10	1.02	ATP transport
1629152_at	*CG31451*	10,674 ± 1916	0.61	0.77	0.83	
1625370_s_at	*gem*	10,661 ± 1147	2.35	20.23	2.02	Transcription factor
1636186_s_at	*Fer2LCH*	10,528 ± 640	1.12	1.17	1.13	Ferritin
1637525_s_at	*Mpcp*	10,468 ± 1136	1.18	0.99	0.97	Phosphate transport
1628694_a_at	14-3-3ε	10,365 ± 565	0.93	0.97	1.08	Protein binding
1625633_a_at	*Argk*	10,229 ± 1768	0.94	1.34	1.06	Arginine kinase
1633443_s_at	*CG2082*	10,203 ± 664	2.01	1.78	1.40	Protein binding
1630452_at	*HDC20239*	9739 ± 2567	0.91	2.49	1.24	
1624564_s_at	*RpS14a*	9683 ± 681	1.22	1.22	1.22	Cytosolic small ribosomal subunit
1634961_s_at	*Vha68-2*	9288 ± 926	9.48	182.70	2.86	V-ATPase A subunit
1634989_at	*Acon*	9255 ± 1106	1.59	1.66	1.38	Aconitate hydratase
1640729_s_at	*nrv3*	9244 ± 2404	0.70	1.00	1.15	Na:K-exchanging ATPase complex
1638351_s_at	*Ef1alpha48D*	9206 ± 663	1.67	3.18	1.21	Translation elongation
1623073_s_at	*ctp*	9178 ± 894	0.99	0.88	1.04	Microtubule motor activity
1625672_s_at	*CT33405*	9074 ± 1,941	0.76	0.92	0.96	
1637393_at	*Vmat*	8981 ± 1797	1.61	1.37	0.96	Monoamine transport
1632117_s_at	*Vha16*	8927 ± 552	0.88	1.10	1.16	V-ATPase C subunit
1625638_a_at	*Hex-A*	8835 ± 1331	2.20	2.25	1.57	Hexokinase
1633034_s_at	*Fer1HCH*	8817 ± 959	1.10	1.19	1.12	Ferritin
1639761_s_at	*Gγ1*	8677 ± 1394	0.80	0.83	0.86	GPCR signaling
1627732_s_at	*CG30415*	8626 ± 913	0.91	1.03	0.91	
1626163_s_at	*Act5C*	8621 ± 1612	0.91	1.18	1.14	Actin
1637059_s_at	*DnaJ-1*	8620 ± 1366	1.38	0.92	1.42	Heat shock protein
1625131_s_at	*Vha55*	8560 ± 862	1.18	1.05	1.00	V-ATPase B subunit
1639596_at	*CG7781*	8555 ± 2131	1.09	1.37	1.58	
1635449_s_at	*Dip-B*	8518 ± 814	11.56	42.13	1.99	Aminopeptidase
1637164_at	*Tctp*	8470 ± 465	1.38	1.62	1.33	Guanyl-nucleotide exchange factor
1625997_s_at	*GH03753*	8393 ± 3436	0.70	2.24	1.26	Transposable element
1631168_at	*CG5687*	8333 ± 963	19.50	432.04	4.80	Sodium:iodide symporter
1639597_at	*Obp44a*	8311 ± 3564	0.39	6.50	0.96	Odorant binding
1633606_s_at	*CG8229*	8224 ± 1617	0.83	0.87	0.84	
1628814_s_at	*CG9894*	8224 ± 2175	0.78	0.79	0.98	
1629659_s_at	*CG5119*	8188 ± 2028	1.19	1.35	1.25	Nucleotide binding

Signals (with standard deviations) represent the average of five replicate normalized expression values. Enrichments in SG relative to brain (B), neurons (N), and all glia (AG) are simple ratios and do not reflect statistically significant differences.

To further investigate the specialized functions of the BBB, we determined the differentially expressed genes in surface glia by performing pairwise comparisons to whole brain, neurons, and all glia using limma software (Smyth, 2004). Table S2 contains differentially expressed genes for all pairwise comparisons performed in limma. Table 2 reports the top 50 enriched surface glia genes for each comparison. Relative to whole brain, there are 1010 genes up-regulated (i.e., enriched) and 3899 genes down-regulated in the surface glia. Relative to neurons, there are 1183 genes up-regulated and 2979 genes down-regulated in surface glia. Relative to all glia, there are 543 genes up-regulated and 568 genes down-regulated in surface glia. Expression fold changes are greatest in the surface glia-neuron comparison (max = 729, mean = 15.6, median = 3.7), followed by the surface glia-brain comparison (max = 247, mean = 6.0, median = 2.9) and the surface glia-all glia comparison (max = 35, mean = 4.5, median = 3.5). This enrichment trend can in part be explained by the amount of surface glia RNA in each comparison sample. Neuronal samples contain no surface glia, brain samples contain a small proportion of surface glia, and all glia samples contain a substantial proportion of surface glia mixed with other glial subtypes. Thus, as expected, the number of differentially expressed genes and their fold changes is maximal in the surface glia-neuron comparison reflecting sample cell-type homogeneity and likely functional specialization.

**Table 2:** **The 50 most enriched surface glia genes according to three separate comparisons**.

**Surface glia/brain**			**Surface glia/neurons**			**Surface glia/all glia**		
**Probe**	**Symbol**	**FC**	**Probe**	**Symbol**	**FC**	**Probe**	**Symbol**	**FC**
1629317_at	*Nplp3*	247	1641566_s_at	*Indy*	729	1625042_at	*CG31288*	35
1629827_s_at	*Hsp70Ba*	144	1639660_s_at	*CG10550*	563	1639694_s_at	*Arc1*	34
1636747_at	*CG8051*	117	1629317_at	*Nplp3*	453	1623624_at	*CG14869*	22
1636947_at	*bbg*	116	1631168_at	*CG5687*	436	1638964_at	*CG7800*	19
1623624_at	*CG14869*	93	1630150_s_at	*Cg25C*	398	1623788_at	*CG7461*	16
1625042_at	*CG31288*	68	1635210_a_at	*Ppn*	367	1639180_at	*Arc1*	16
1633428_at	*prc*	67	1631925_at	*Mdr65*	299	1635522_a_at	*santa-maria*	14
1631380_s_at	*LanB1*	66	1636835_at	*CG16700*	292	1628075_at	*olf186-M*	14
1624982_s_at	*CG5080*	55	1634767_at	*CG6126*	275	1628779_a_at	*svp*	14
1630142_at	*sog*	46	1641320_s_at	*CG3168*	241	1631535_at	*Vinc*	13
1641490_s_at	*Tsp*	46	1636274_at	*wgn*	225	1640065_at	*GstE7*	13
1628235_at	*CG7203*	42	1633674_at	*cv-d*	216	1632021_at	*Cyp6a20*	13
1632317_at	*CG3036*	41	1639229_at	*vkg*	215	1638661_at	*yip7*	13
1626839_s_at	*blot*	40	1640509_s_at	*lama*	213	1640303_a_at	*pst*	13
1639424_at	*CG6910*	38	1634961_s_at	*Vha68-2*	203	1630633_at	*CG13130*	12
1634620_a_at	*stl*	38	1636947_at	*bbg*	203	1627073_a_at	*CG10126*	12
1628075_at	*olf186-M*	37	1635183_at	*Spn43Ab*	180	1624543_s_at	*–*	12
1635210_a_at	*Ppn*	36	1630380_at	*CG3837*	154	1636947_at	*bbg*	12
1630233_at	*CG5399*	36	1624839_at	*h*	152	1629062_at	*CG13252*	12
1640509_s_at	*lama*	35	1633639_at	*Cyp28d1*	152	1626271_at	*CG9634*	12
1625116_at	*trol*	35	1636747_at	*CG8051*	148	1624744_a_at	*nuf*	12
1641566_s_at	*Indy*	34	1627773_a_at	*Jheh2*	146	1638226_at	*CG10562*	11
1629256_a_at	*CG31547*	34	1626839_s_at	*blot*	142	1625476_a_at	*CG1674*	11
1638661_at	*yip7*	33	1631569_s_at	*Gp150*	139	1625477_a_at	*CG4797*	11
1634767_at	*CG6126*	33	1623624_at	*CG14869*	135	1628235_at	*CG7203*	11
1625477_a_at	*CG4797*	33	1623200_at	*fng*	133	1636149_at	*CG31705*	11
1628739_at	*Vmat*	32	1624982_s_at	*CG5080*	129	1637481_at	*Tollo*	11
1624156_at	*Ugt86Da*	31	1633112_at	*moody*	127	1631948_s_at	*Clk*	10
1623364_at	*CG4250*	29	1625477_a_at	*CG4797*	116	1638132_at	*CG10184*	10
1626645_at	*CG8596*	28	1631646_at	*spri*	116	1637281_at	*–*	10
1639660_s_at	*CG10550*	28	1639424_at	*CG6910*	104	1636274_at	*wgn*	10
1623655_at	*Yp2*	27	1629009_at	*Cyp28a5*	100	1629009_at	*Cyp28a5*	10
1640912_s_at	*scaf*	26	1628465_a_at	*CG34417*	97	1626319_a_at	*IM10*	10
1633674_at	*cv-d*	25	1640912_s_at	*scaf*	96	1633254_at	*CG7787*	10
1628052_at	*Cyp6a17*	25	1636653_at	*nec*	94	1640922_a_at	*Hnf4*	9
1632160_s_at	*CG15279*	24	1631380_s_at	*LanB1*	94	1640896_at	*CG4462*	9
1640896_at	*CG4462*	24	1634063_a_at	*stumps*	93	1636747_at	*CG8051*	9
1635770_at	*CG31097*	23	1638964_at	*CG7800*	90	1634237_at	*nimC3*	9
1627000_s_at	*CG6231*	23	1623364_at	*CG4250*	88	1624156_at	*Ugt86Da*	9
1626724_at	*CG32687*	23	1637359_at	*Lsd-2*	86	1624839_at	*h*	9
1623019_a_at	*Unc-89*	23	1632500_at	*CG10226*	85	1627315_s_at	*Pld*	9
1625041_at	*Oatp74D*	21	1632980_at	*CG3902*	85	1633765_at	*–*	9
1636268_at	*CG10570*	21	1632744_a_at	*if*	83	1625185_at	*CAH2*	9
1629271_at	*CG10444*	21	1632317_at	*CG3036*	83	1631227_at	*CG5793*	9
1638956_at	*Fas2*	20	1632839_a_at	*CG8312*	81	1637089_at	*Syb*	9
1631168_at	*CG5687*	20	1640303_a_at	*pst*	79	1630286_at	*AnnIX*	9
1629062_at	*CG13252*	20	1632021_at	*Cyp6a20*	78	1632160_s_at	*CG15279*	9
1625857_at	*CG8451*	20	1629886_s_at	*for*	78	1641566_s_at	*Indy*	9
1631359_s_at	*Vha100-2*	19	1638063_at	*CG34417*	74	1635770_at	*CG31097*	8
1634036_at	*CG8788*	19	1636149_at	*CG31705*	73	1640703_at	*CG30460*	8

Fold changes (FC) are limma-estimated, and all genes are statistically significant according to an FDR-adjusted P < 0.05.

### Validating the surface glia transcriptome as a BBB gene profile

To infer specialized molecular pathways present in the surface glia from their transcriptome, we performed gene set enrichment analyses using DAVID Bioinformatics (Huang et al., 2009a,b). Table 3 lists selected enriched Gene Ontology (GO) categories, KEGG pathways, Interpro domains, and PIR superfamilies among genes enriched in surface glia relative to brain, neurons, and all glia (for all results see Table S3). The DAVID Bioinformatics results support the view of the surface glia being the primary component of the *Drosophila* BBB.

**Table 3:** **Selected results from gene set enrichment analyses performed using DAVID Bioinformatics on differentially expressed genes enriched in surface glia (SG) relative to brain (B), neurons (N), and all glia (AG)**.

**Enriched term**	**Category**	**SG/B**		**SG/N**		**SG/AG**	
		**N**	**P (adj)**	**N**	**P (adj)**	**N**	**P (adj)**
**DRUG METABOLISM**							
dme00040:Pentose and glucuronate interconversions	KEGG pathway			12	4.6E-02		
dme00980:Metabolism of xenobiotics by cytochrome P450	KEGG pathway			20	1.6E-03		
GO:0004364 glutathione transferase activity	GO MF			11	3.2E-02		
**CELL ADHESION MOLECULES**							
GO:0005912 adherens junction	GO CC	14	1.1E-04	14	1.1E-03		
GO:0007155 cell adhesion	GO BP	22	2.8E-02	30	8.5E-04	21	4.5E-03
**TRANSPORTERS**							
GO:0015293 symporter activity	GO MF	16	2.4E-02	19	8.4E-03	14	1.4E-02
GO:0015849 organic acid transport	GO BP			12	4.7E-02		
IPR001140:ABC transporter, transmembrane region	Interpro domain	8	5.0E-02				
IPR011701:Major facilitator superfamily MFS-1	Interpro domain	20	1.6E-02	21	2.8E-02	16	1.9E-02
Transport	SP_PIR keyword			49	1.0E-02	31	1.8E-02
**BASEMENT MEMBRANE/ECM**							
GO:0005605 basal lamina	GO CC	5	1.4E-03	5	3.3E-03		
GO:0031012 extracellular matrix	GO CC	18	5.5E-07	19	2.4E-06	11	4.1E-03
**METABOLISM**							
dme00071:Fatty acid metabolism	KEGG pathway	11	1.2E-02	14	2.3E-03	9	2.2E-02
GO:0016052 carbohydrate catabolic process	GO BP			23	4.1E-06		
**MISCELLANEOUS**							
GO:0001666 response to hypoxia	GO BP	7	3.0E-02				
GO:0005811 lipid particle	GO CC	39	6.2E-06	73	2.4E-21	23	1.9E-02
GO:0006952 defense response	GO BP	22	2.8E-02	26	2.3E-02		
GO:0022626 cytosolic ribosome	GO CC			31	1.2E-09		
GO:0045185 maintenance of protein location	GO BP	12	1.5E-03				
IPR015897:CHK kinase-like	Interpro domain			14	3.7E-02	10	4.2E-02
PIRSF036514:alpha-crystallin-related small heat shock protein	PIR superfamily	6	6.7E-03				

GO MF, Gene ontology molecular function; BP, biological process; and CC, cellular component. P-values are Benjamini-adjusted.

Consistent with the surface glia being a chemical protection interface, we see numerous enriched categories associated with drug metabolism, cell adhesion, and transport (Table 3). Selected genes in these categories are listed in Table 4 and reveal striking signatures of chemical protection physiology. For example, there are numerous cytochrome P450 (CYP), glutathione S-transferase (GST), and UDP-glucuronosyltransferase (UGT) enzymes enriched in the surface glia. These enrichment results account for all phases of drug metabolism. Phase I reactions include oxidation reactions by CYPs; phase II reactions include conjugation reactions, such as glucoronidation by UGTs and glutathionylation by GSTs; and phase III reactions involve excretion of drug metabolites by transporters (Sheweita, 2000; Homolya et al., 2003). These excretory transporters are often ABC transporters. Notable ABC transporters involved in drug metabolism and efflux include members of the B and C class, such as ABCB1 and MRP1-4 (ABCC1-4). Our results show three B class ABC transporters to be highly enriched in the surface glia (*Mdr65*, *Mdr49*, and *CG10226*). These three transporters are all highly homologous to vertebrate ABCB1, and we have previously shown that Mdr65 has a conserved function in drug efflux at the apical surface of the *Drosophila* BBB (Mayer et al., 2009). Other enriched transporters in the surface glia include numerous SLC transporters, reflecting the function of the surface glia as a metabolic barrier similar to the vertebrate vascular endothelial BBB component.

**Table 4:** **Differentially expressed genes enriched in surface glia (SG) relative to brain (B), neurons (N), and all glia (AG) involved in characteristic BBB structures (the basal lamina) and physiologies (drug metabolism, cellular junctions, and transport)**.

**Probe**	**Symbol**	**SG/B**	**SG/N**	**SG/AG**	**Function**
**BASAL LAMINA/ECM**					
1630150_s_at	*Cg25C*	7.01	398.29	2.37	Collagen
1635210_a_at	*Ppn*	35.65	366.65	2.57	Structural constituent
1639229_at	*vkg*	8.88	215.02		Collagen
1623624_at	*CG14869*	93.06	135.34	21.93	Basal lamina/ECM
1633428_at	*prc*	67.36	67.61		Collagen-like
1641324_at	*LanA*	18.52	24.42		Laminin
1632666_at	*LanB2*	7.09	16.07		Laminin
**DRUG METABOLISM**					
1633639_at	*Cyp28d1*		151.51		Cytochrome P450
1629009_at	*Cyp28a5*		100.38	9.68	Cytochrome P450
1632021_at	*Cyp6a20*	10.84	78.07	12.72	Cytochrome P450
1624156_at	*Ugt86Da*	30.74	72.63	9.26	UDP-glucuronosyltransferase
1635727_a_at	*Cyp4g15*		42.62	3.45	Cytochrome P450
1623957_s_at	*GstS1*		38.55		Glutathione S-transferase
1640065_at	*GstE7*	13.84	20.65	12.98	Glutathione S-transferase
1628345_at	*Cyp6a9*	7.11	18.98		Cytochrome P450
1639944_at	*Cyp9f2*		18.69	3.83	Cytochrome P450
1628052_at	*Cyp6a17*	24.54	13.35		Cytochrome P450
1641191_s_at	*Ugt36Bc*	4.54	12.01	5.16	UDP-glucuronosyltransferase
1637129_at	*GstE3*	6.17	11.89	4.84	Glutathione S-transferase
1637309_a_at	*Cyp12e1*	1.89	10.52	4.23	Cytochrome P450
1641481_at	*Ugt86Dd*	4.24	6.63	2.65	UDP-glucuronosyltransferase
1628353_at	*GstD1*	2.01	5.56	2.24	Glutathione S-transferase
1632070_at	*Ugt58Fa*	2.68	4.63	2.04	UDP-glucuronosyltransferase
1639381_at	*Cyp6a19*	4.72	4.31	1.98	Cytochrome P450
**SEPTATE JUNCTIONS**					
1633112_at	*moody*		127.46		GPCR signaling
1624774_a_at	*Fas2*	4.68	11.58	3.60	Homophilic adhesion
1627491_at	*G-iα65A*		11.23		GPCR signaling
1637410_s_at	*l(2)gl*	3.77	10.09	6.20	Linker
1627651_a_at	*loco*	6.76	8.69		GPCR signaling
1636146_at	*Nrg*		5.63	5.40	Heterophilic adhesion
1625215_s_at	*nrv2*		3.45		Na,K-ATPase
**TRANSPORT**					
1641566_s_at	*Indy*	34.46	729.03	8.52	SLC13—Na-dep. sulfate/carboxylate transport
1631168_at	*CG5687*	20.28	435.65	5.21	SLC5—sodium:iodide symport
1631925_at	*Mdr65*	7.59	298.68	2.97	MDR/ABC-B
1636835_at	*CG16700*	9.23	292.19	2.56	SLC36—H^+^-coupled amino acid transport
1634767_at	*CG6126*	33.08	275.36	2.48	SLC22—organic anion transport
1641320_s_at	*CG3168*	4.16	241.14	5.92	Putative sugar transport
1636747_at	*CG8051*	117.32	147.84	9.34	SLC16—monocarboxylate transport
1626839_s_at	*blot*	39.99	141.76	6.01	Neurotransmitter transport
1625477_a_at	*CG4797*	32.57	116.05	11.00	SLC2—sugar transport
1637359_at	*Lsd-2*	2.96	85.98	3.05	Lipid transport
1632500_at	*CG10226*	7.86	85.48	4.97	MDR/ABC-B
1628739_at	*Vmat*	32.04	41.26		SLC18—monoamine transport
1628659_at	*Mdr49*	12.52	33.67		MDR/ABC-B
1629271_at	*CG10444*	20.73	22.65	3.06	SLC5—sodium:iodide symport

Enrichment values are limma-estimated fold changes, and all genes are significant according to an FDR-adjusted P < 0.05.

In compliance with the BBB functioning as a diffusion barrier, numerous genes constituting SJs are enriched in the surface glia, including the components *Fasciclin 2* (*Fas2*), *lethal (2) giant larvae* (*l(2)gl*), *Neuroglian* (*Nrg*), and *nervana 2* (*nrv2*) (Table 4). Also enriched is Moody, a GPCR involved in a signaling pathway that regulates SJ formation (Bainton et al., 2005). Interestingly, the innexin gap junction genes *ogre (inx1)* and *inx2* are enriched in the surface glia and *Drosophila inx1* and *inx2* have recently been linked to coordination of neural stem cell proliferation in response to the metabolic status of the animal (Spéder and Brand, 2014). Thus, the surface glia transcriptome may provide insight into higher order BBB processes. Overall, gene set enrichment analyses of the surface glia transcriptome confirms that the surface glia possess characteristics of BBB physiology.

Although the expression and large enrichment of genes listed in Table 4, especially *moody* and *Mdr65*, indicate that our cell isolation techniques are of high purity, we wanted to further validate our approach. Therefore, we searched the literature for genes known to be expressed and functional in the surface glia. We selected 29 genes represented by 36 probe IDs (listed in Table 5). We primarily focused on genes where mutations and/or RNAi-mediated knockdown lead to BBB leakiness indicating that the expressed genes function to maintain BBB integrity. If our surface glia transcriptome is valid, we would expect these 29 genes to be present in our data. To decide whether a gene is expressed, we look at two values: (1) the average normalized expression level of five replicate samples and (2) the sum of five replicate sample Present calls for the gene's probe(s). While there are no standards for calling a gene expressed based on Affymetrix data, we set a threshold of a Present sum ≥ 4 and/or an expression level >100. For example, *moody* and *Mdr65*, two genes enriched in the surface glia, have average normalized expression levels of 2412 and 6078 units, respectively. Both genes also have Present call sums of 5, meaning they were called Present in all 5 replicates of sorted surface glia samples. According to this metric, most of the genes in Table 5 can be classified as being expressed in our samples of surface glia. However, our microarray methods did not recognize expression of the following known surface glia genes (Present sum < 4 AND expression < 100): *sinuous (sinu)*, *double parked* (*dup*), and *Gliotactin* (*Gli*). In addition, it is not clear via Affymetrix GeneChips data whether the following genes are expressed in surface glia (Present sum < 4 OR expression < 100): *Neu3*, *scrib*, *vari*, and *coiled* (*cold*). However, preliminary data from deep sequencing of the surface glia transcriptome indicate that the above undetected genes are expressed in the BBB glia, and that *dup*, *Gli*, *Neu3*, *scrib*, and *vari* are also enriched in the BBB glia (data not shown). This suggests that although we can be reasonably confident in the genes present in our surface glia microarray, we need to be cautious in our interpretation of the absent calls, as these could be false negatives.

**Table 5:** **Gene expressed in surface glia at any stage of development according to the literature**.

**Probe**	**Symbol**	**Stage**	**Glia**	**SG signal**	**P**	**SG/B**	**SG/N**	**SG/AG**	**Pheno**	**References**
1628276_s_at	*Bsg*	A	sg	7831 ± 851	5	8.12	5.78	2.23		Curtin et al., 2007; Edwards and Meinertzhagen, 2010
1627454_a_at	*cora*	E	spg	7100 ± 887	5	1.74			X	Stork et al., 2008
1631925_at	*Mdr65*	A	spg	6078 ± 1406	5	7.59	298.68	2.97	X	Mayer et al., 2009
1640457_s_at	*Bsg*	A	sg	5866 ± 1154	5	−1.57	2.51	3.29		Curtin et al., 2007; Edwards and Meinertzhagen, 2010
1641566_s_at	*Indy*	A	pg	5716 ± 1370	5	34.46	729.03	8.52	X	DeSalvo et al., 2011
1632465_s_at	*CG6424*	A	pg	4878 ± 1002	5					DeSalvo et al., 2011
1625215_s_at	*nrv2*	EL	spg	3026 ± 667	5		3.45		X	Stork et al., 2008
1636146_at	*Nrg*	EL	spg	2645 ± 914	5		5.63	5.40	X	Stork et al., 2008; Hatan et al., 2011
1633112_at	*moody*	A	spg	2412 ± 824	5		127.46		X	Bainton et al., 2005; Schwabe et al., 2005
1624021_a_at	*dlg1*	L	spg	2241 ± 1521	5	−2.60	−2.48		X	Unhavaithaya and Orr-Weaver, 2012
1627651_a_at	*loco*	A	sg	2165 ± 1139	5	6.76	8.69		X	Schwabe et al., 2005; Kaplow et al., 2008
1629844_s_at	*rap*	L	sg	1290 ± 653	5		−2.26			Kaplow et al., 2008
1637463_a_at	*Nrg*	EL	spg	1099 ± 868	5		4.52		X	Stork et al., 2008; Hatan et al., 2011
1627114_at	*CG3793*	A	spg	1095 ± 379	5				X	DeSalvo et al., 2011
1626001_at	*Nrg*	EL	spg	991 ± 700	3				X	Stork et al., 2008; Hatan et al., 2011
1628336_a_at	*Lac*	E	sg	839 ± 296	5				X	Strigini et al., 2006
1623571_a_at	*CG9328*	A	pg	738 ± 537	4	−2.97			X	DeSalvo et al., 2011
1627491_at	*G-iα65A*	E	spg	697 ± 824	4		11.23		X	Schwabe et al., 2005
1628262_a_at	*CG1322*	E	sg	459 ± 604	5					Layden et al., 2006
1628135_s_at	*pck*	E	spg	421 ± 391	4				X	Stork et al., 2008
1641247_at	*Ranbp21*	A	pg	356 ± 413	5				X	DeSalvo et al., 2011
1632164_at	*scrib*	L	spg	336 ± 314	3		−5.91			Hatan et al., 2011
1635984_at	*Neu3*	A	spg	225 ± 49	1					DeSalvo et al., 2011
1639459_a_at	*Nrx-IV*	EL	spg	213 ± 168	4				X	Baumgartner et al., 1996; Schwabe et al., 2005; Strigini et al., 2006; Hatan et al., 2011
1639402_a_at	*scrib*	L	spg	191 ± 119	3					Hatan et al., 2011
1637017_at	*Cont*	E	spg	167 ± 181	4	−4.35			X	Stork et al., 2008
1631573_a_at	*wunen*	E	spg	166 ± 138	4	−3.31			X	Ile et al., 2012
1639768_at	*coiled*	EL	spg	152 ± 210	3	−5.87	−10.20		X	Hijazi et al., 2011; Syed et al., 2011
1633904_at	*scrib*	L	spg	151 ± 122	2	−3.60	−4.07			Hatan et al., 2011
1623874_at	*CG14215*	A	pg	150 ± 127	4				X	DeSalvo et al., 2011
1625358_s_at	*vari*	A	pg	144 ± 88	2	−2.05			X	DeSalvo et al., 2011
1629115_s_at	*scrib*	L	spg	96 ± 75	3	−3.80	−7.27			Hatan et al., 2011
1627649_at	*Neu3*	A	spg	43 ± 16	0	−3.29				DeSalvo et al., 2011
1637014_at	*sinu*	E	spg	30 ± 15	1	−19.50	−22.60	−8.78	X	Stork et al., 2008
1624994_at	*dup*	L	spg	19 ± 5	2					Unhavaithaya and Orr-Weaver, 2012
1624203_s_at	*Gli*	A	spg	8 ± 3	1	−2.18			X	DeSalvo et al., 2011

We found 29 genes for which 36 probes are listed here—genes are often represented by multiple probes (e.g., Nrg). For “Stage,” E, embryo; L, larvae; and A, adult. The “Glia” column specifies which glia layer the gene is expressed in—sg, both surface glia layers; spg, subperineurial glia; and pg, perineurial glia. Signals (with standard deviations) represent the average of five replicate normalized expression values. P = the P sum, or the number of replicates (max = 5) for which the probe was called present. Enrichment values are limma-estimated fold changes significant at an FDR-adjusted P < 0.05. An X in the “Pheno” column denotes that disrupting expression or function of the gene produces a BBB phenotype—usually leakage of large MW dextrans into the brain.

**Figure 4:**
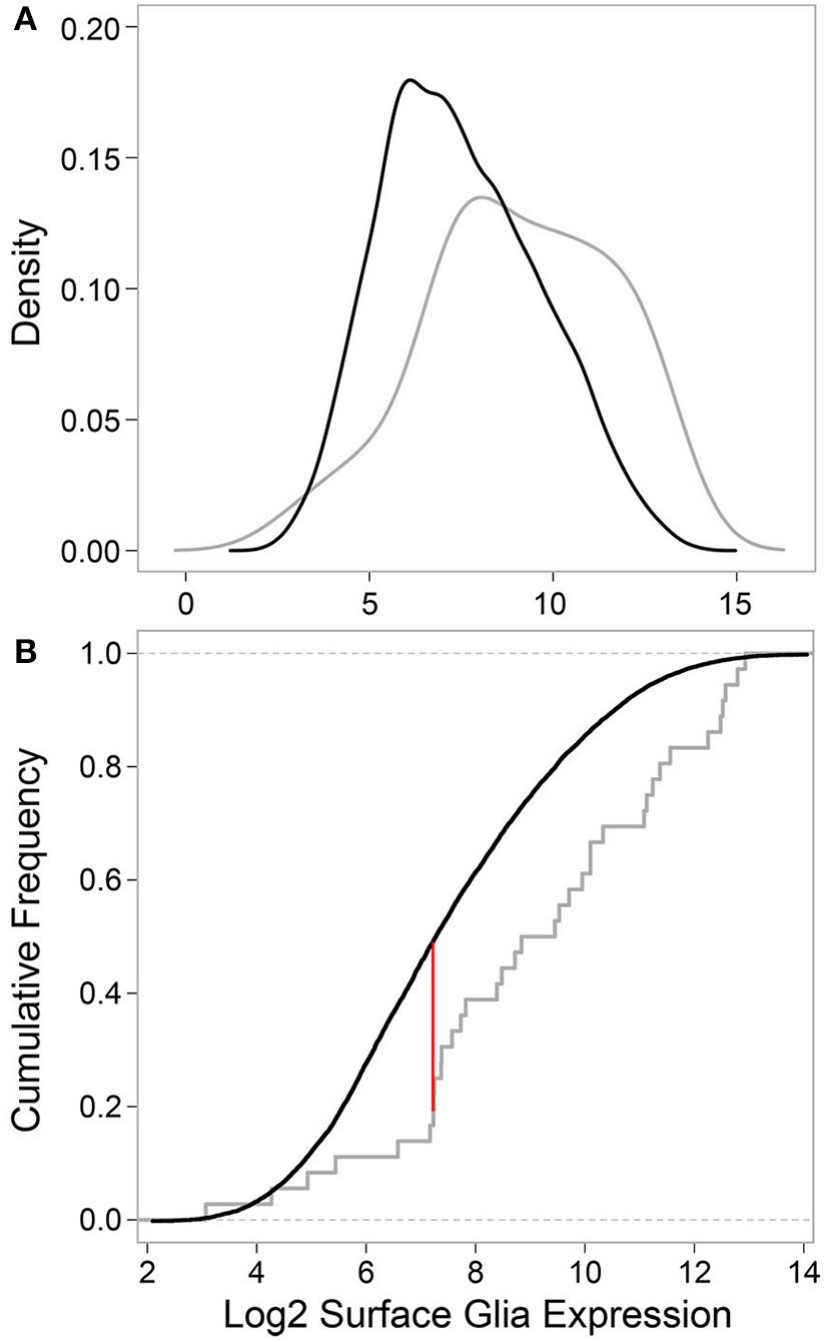
**Comparing data for known surface glia genes to all other probes on the microarray validates the surface glia transcriptome. (A)** Kernal density estimates based on surface glia log2 gene expression for known surface glia genes (gray) and all other probes on the microarray (black). The distribution of data for known surface glia genes is significantly greater than that for all other probes on the microarray (Two-sample Kolmogorov-Smirnov test, *D* = 0.3435, *P* = 0.0004). **(B)** A cumulative frequency plot of the same data also illustrates the significant difference in distributions between known surface glia genes and all other probes on the microarray. The red line corresponds to the D-statistic, the point of greatest separation between the curves. At this point, 81.6% of known surface glia genes have higher expression levels, which is in stark contrast to 51.7% for all other probes on the microarray.

Next, we tested whether the 29 surface glia genes, as a group, have greater expression levels and Present sums compared to all other genes on the microarray. With respect to gene expression level, a two-sample Kolmogorov-Smirnov test revealed a significant difference in the distribution of expression values for known surface glia genes and all other genes on the microarray (*D* = 0.3442, *p* = 0.0004). This difference can be seen graphically in Figure 4: a density plot (Figure 4A) and a cumulative frequency plot (Figure 4B) of log2 surface glia expression for both groups of genes showing that, by and large, known surface glia genes have greater expression values than all other genes on the microarray. With respect to Present sum data, we used a hypergeometric test to determine whether the distribution of Present sums in known surface glia genes was significantly different (and greater) than that for all other genes on the microarray. According to our stringent standard of requiring a Present sum ≥4 to call a probe expressed, we find that 24 of 36 (67%) probes representing known surface glia genes have a Present sum ≥4. Comparing this to 4488 of 11645 (39%) of the remaining probes on the array, the hypergeometric test indeed finds a significant difference between these distributions (*p* = 0.0002). Even if we loosen our criteria and allow a Present sum ≥3, there is still a significant difference (29 of 36 [81%] for known surface glia genes and 6063 of 11645 [52%] for all other probes—*p* = 9.5 × 10^−5^). Overall, we find that our microarray data shows positive expression for known surface glia genes leading us to conclude that the surface glia transcriptome is highly quantitative and accurate.

**Figure 5:**
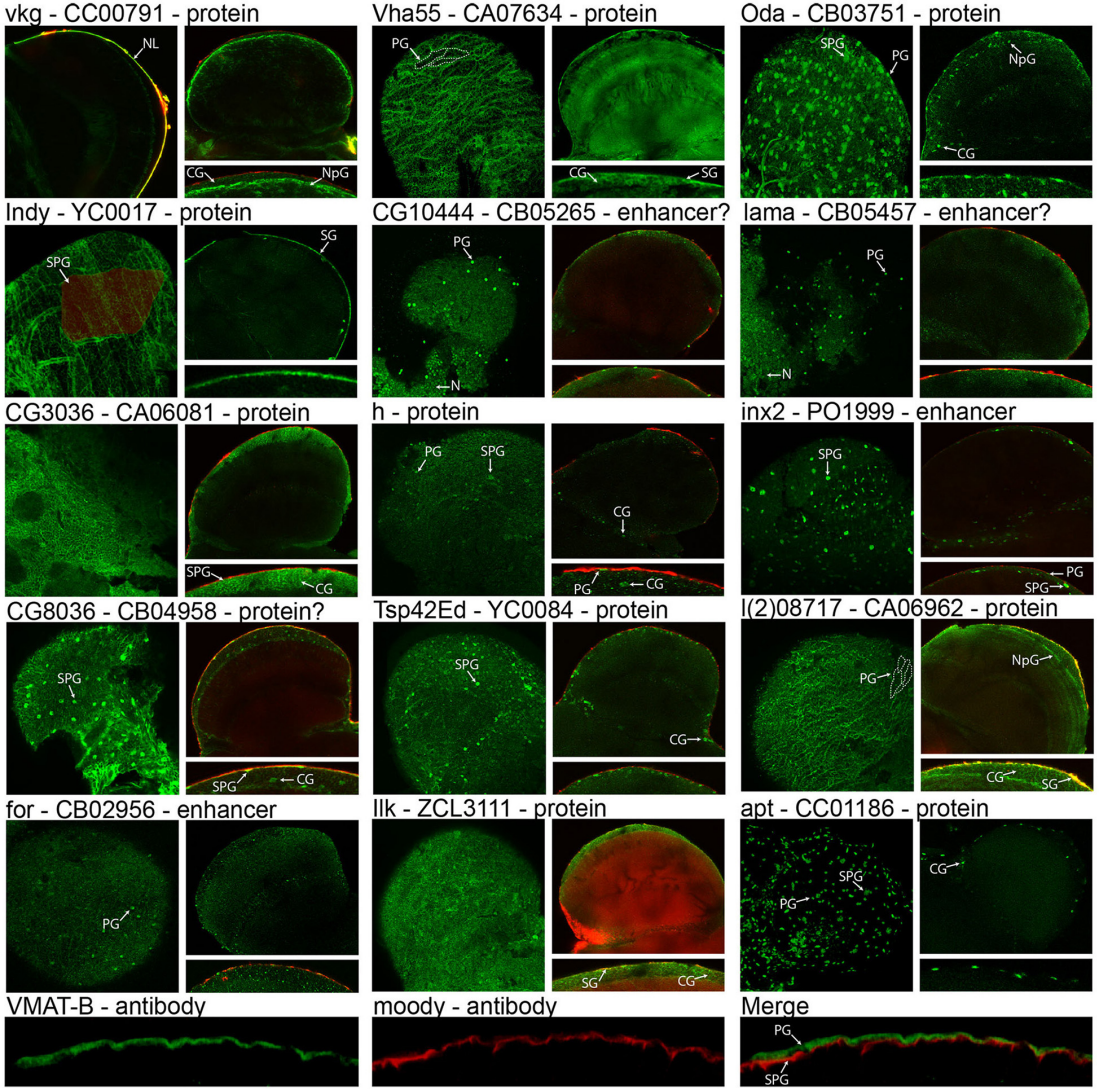
**Expression and localization of surface glia-enriched genes**. Gene symbols are followed by the FlyTrap database ID number and whether the GFP exon results in a GFP-fusion protein (Protein), likely results in a GFP-fusion protein (Protein?), is controlled by nearby enhancers (Enhancer), or is likely controlled by nearby enhancers (Enhancer?). With the exception of *vkg*, each FlyTrap line has a surface image followed by a cross section image and a zoomed-in image of the surface glia layer in cross section. For *vkg*, instead of a surface image, we present a cross-section showing the GFP-positive neural lamella. Localization to the surface glia layer can be assessed using surface images that show membrane GFP at the boundaries of perineurial glia (PG) [see dotted lines in *Vha55* and *l(2)08717*] or subperineurial glia (SPG) (see shaded cell in *Indy*). In cross section images of membrane GFP, localization to the surface glia can be determined because injection with 70 kDa rhodamine dextran demarcates the PG layer (no dextran was used for *Vha55*, *Oda*, *apt*, and VMAT-B). For nuclear GFP, surface images reveal small PG nuclei and large SPG nuclei. In cross section, these nuclei are within the dextran layer (PG-localized) or slightly below (SPG-localized). Many surface glia-enriched genes are also expressed in other glial subtypes, such as cortex glia (CG) and neuropil glia (NpG), and in rare cases we found that surface glia-enriched genes were also expressed in neurons (N). VMAT-B expression is visualized with polyclonal antibodies (green) and localized by co-staining with antibodies to the SPG-specific Moody protein (red).

Central to validating our cell isolation and transcriptomic techniques is showing that genes identified as enriched in the surface glia are indeed expressed in these cell layers. To demonstrate surface glia-localized expression, we searched the FlyTrap GFP Protein Trap database (http://flytrap.med.yale.edu/) (Morin et al., 2001; Kelso et al., 2004; Buszczak et al., 2007; Quinones-Coello et al., 2007) for protein and enhancer traps available for surface glia-enriched genes. As a control, we also included one gene (*Vha55*) that, based on our transcriptome, was roughly equally expressed in neurons, glia, and surface glia. We also searched the Bloomington Stock Collection for transgenic lines containing GFP-fusion proteins for any surface glia-enriched genes, and found one for *hairy* (*h*). Lastly, we targeted the expression of VMAT using polyclonal antibodies specific to the glia-specific isoform of VMAT (DVMAT-B) previously found to store histamine in the *Drosophila* visual system (Romero-Calderon et al., 2008). Microarray data for all genes in Figure 5 are listed in Table S4. The images in Figure 5 indeed demonstrate that surface glia-enriched genes identified in the microarray data are expressed in these cell layers. However, surface glia enrichment does not equate to specific expression in the surface glia. Many surface glia-enriched genes are also expressed in other glial subtypes. For example, the first gene shown in Figure 5 is *viking* (*vkg*), which encodes a collagen IV protein, and is enriched 9-fold relative to whole brain and 215-fold relative to neurons. The GFP protein trap for *vkg* shows pan-glial expression (see also Figure 1 for another image of the *vkg* protein trap), which is consistent with a previous study (Freeman et al., 2003). Interestingly, the *vkg* protein trap also allows for visualization of the neural lamella, and its enrichment in surface glia suggests deposition of the neural lamella by the surface glia.

Surface glia-localized expression can be assessed using both brain surface and cross-section confocal images (Figure 5). Co-localization with injected 70 kDa dextran helps to differentiate the PG from the SPG since dextran demarcates the PG layer (DeSalvo et al., 2011). Expression in the surface glia can be identified by any of the following characteristics: (1) surface images show a “flagstone” pattern of cell junctions characteristic of the PG, e.g., *Vha55*, *I'm not dead yet* (*Indy*), and *l(2)08717*; (2) surface images show the SJ boundaries characteristic of the SPG, e.g., *Indy*; (3) surface and cross-section images show numerous, small PG nuclei, e.g., *Ornithine decarboxylase antizyme* (*Oda*), *CG10444*, *innexin 2* (*inx2*), *lamina ancestor* (*lama*), *foraging* (*for*), and *apontic* (*apt*); and (4) surface and cross-section images show sparse, large SPG nuclei, e.g., *Oda*, *CG8036*, *h*, *inx2*, and *apt*. Furthermore, many of the images (e.g., *Vha55*, *Indy*, *CG3036*, and *CG8036*) show a pattern of GFP expression similar to that seen when staining adult brains with antibodies to the Moody protein (Schwabe et al., 2005; Mayer et al., 2009). This pattern resembles a mosaic of small circular cell junctions, which is due to either (1) contact between the basal surface of the SPG and the underlying cortex glia, or (2) expression in both the SPG and cortex glia. Indeed, many of the surface glia-enriched genes are also expressed in the cortex glia. Membrane-bound GFP expression in cortex glia is seen in *CG3036*, *CG8036*, *l(2)08717*, and *Integrin-linked kinase* (*Ilk*). Nuclear-GFP expression in cortex glia is seen in *Oda*, *h*, *Tetraspanin 42Ed* (*Tsp42Ed*), and *apt*.

Regarding expression of the glia-specific DVMAT-B, we co-stained adult brains with Moody and DVMAT-B antibodies. Moody is specifically expressed in the SPG (Bainton et al., 2005; Mayer et al., 2009), and the results show DVMAT-B expression apical and non-overlapping with Moody, thus pinpointing DVMAT-B to the PG layer (Figure 5). Of the 16 surface glia-enriched genes shown in Figure 5, we found that *Vmat* and *Indy* were the only surface glia-specific genes in the adult brain. Furthermore, we also found that one of the protein traps (*Ilk* GFP) caused the BBB to be leaky, indicated by the large accumulation of 70 kDa dextran in the brains. *Ilk* is expressed in surface and cortex glia. In this case, GFP fusion likely disrupts protein function leading to BBB leakiness. In addition to showing that surface glia-enriched genes are indeed expressed in these cell types, we also found that our control gene *Vha55* had a more global expression pattern in the brain. This is consistent with our transcriptome, further confirming the validity of our cell isolation and transcriptomic methods. Thus, these data provide a suitable starting point for an investigator interested in elucidating gene function in surface glia-localized processes.

While little is known about the fly surface glia, there is a wealth of knowledge on neuronal physiology where many proteins and processes are highly conserved among metazoans (Venter et al., 1988; Anderson and Greenberg, 2001). Thus, we can further validate our cell isolation and transcriptomic methods by analyzing neuronal genes and pathways. As anticipated, overrepresented gene set enrichment categories related to neuronal physiology are seen for genes enriched in whole brains, neurons, and all glia samples relative to those of the surface glia (Table 6). Neurons are 3-fold more numerous than glia in the fly brain (Pfrieger and Barres, 1995), thus, it was to be expected that the genes enriched in our whole brain and neuron samples largely function in synaptic transmission, axonogenesis, vesicle-mediated transport, regulation of neurotransmitter levels, and neurotransmitter receptor activity. Genes enriched in all glia relative to the surface glia include those involved in axon guidance, neuron development, and synapse formation. These functions are consistent with the activities of the abundant cortex and neuropil glia, which are more intimately involved in neuronal development and function (Edwards and Meinertzhagen, 2010).

**Table 6:** **Selected results from gene set enrichment analyses performed using DAVID Bioinformatics on differentially expressed genes enriched in brain (B), neurons (N), and all glia (AG) relative to surface glia (SG)**.

**Enriched term**	**Category**	**SG/B**		**SG/N**		**SG/AG**	
		**N**	**P (adj)**	**N**	**P (adj)**	**N**	**P (adj)**
GO:0001505 regulation of neurotransmitter levels	GO BP	55	8.0E-07	32	2.2E-02		
GO:0007268 synaptic transmission	GO BP	90	6.7E-10	61	3.8E-06		
GO:0007409 axonogenesis	GO BP	80	1.3E-05	76	9.1E-12	17	1.4E-02
GO:0007411 axon guidance	GO BP	58	8.4E-05	57	2.2E-10	13	2.8E-02
GO:0007610 behavior	GO BP	129	2.5E-02	106	1.1E-04		
GO:0007611 learning or memory	GO BP	32	2.4E-02	26	8.0E-03		
GO:0008038 neuron recognition	GO BP			23	1.1E-04		
GO:0008355 olfactory learning	GO BP			18	2.2E-02		
GO:0016192 vesicle-mediated transport	GO BP	136	1.8E-05	85	4.2E-02		
GO:0016319 mushroom body development	GO BP			20	5.2E-03		
GO:0019933 cAMP-mediated signaling	GO BP	11	3.3E-02	11	1.6E-03		
GO:0030594 neurotransmitter receptor activity	GO MF	32	3.7E-02	29	3.9E-04		
GO:0031644 regulation of neurological system process	GO BP			9	2.4E-02		
GO:0034702 ion channel complex	GO CC	27	5.3E-03	26	6.2E-06	9	1.0E-02
GO:0045202 synapse	GO CC	65	6.4E-08	53	3.7E-09		
GO:0048512 circadian behavior	GO BP	22	1.5E-03	17	4.3E-03		
GO:0048666 neuron development	GO BP	131	2.9E-07	114	1.0E-12	26	2.9E-03

GO BP, Gene ontology biological process; MF, molecular function; and CC, cellular component. P-values are Benjamini-adjusted.

### Adult BBB transcriptome points to genes required throughout development for BBB function

Having shown that our adult BBB transcriptome is a reliable resource, we were now well positioned to ask how the adult BBB profile compares to that of the embryo. We used data from the Berkeley *Drosophila* Genome Project (BDGP) *in situ* database (http://insitu.fruitfly.org/cgi-bin/ex/insitu.pl) to obtain gene expression patterns in the embryonic CNS. The BDGP *in situ* database classifies *in situ* gene expression patterns during embryonic development according to a controlled vocabulary that corresponds to specific anatomic structures (Tomancak et al., 2002, 2007). The BDGP vocabulary includes gene expression in the lateral cord surface glia and central brain surface glia for embryos in stages 13–16. We asked whether surface glia-enriched genes in the adult brain are more likely to have embryonic surface glia *in situ* staining compared to non-surface glia-enriched genes. Indeed, we found that genes enriched in adult surface glia relative to whole brain are more likely to contain embryonic surface glia *in situ* staining (Chi-squared test with Yates' continuity correction: *X*^2^ = 4.76, *df* = 1, *p*-value = 0.0291, odds ratio = 2.12). We also obtained a significant result for genes enriched in surface glia relative to neurons (*X*^2^ = 7.32, *df* = 1, *p*-value = 0.0068, odds ratio = 2.26). The results point to 18 genes that are expressed in surface glia during all stages of development and adulthood (*in situ* images can be found on the BDGP website). These genes include: (1) six known glial genes (*G-iα65A*, *Mdr65*, *Glutamine synthetase 2*, *repo*, *moody*, and *nrv2*) with previously published *in situ* images (Xiong et al., 1994; Auld et al., 1995; Freeman et al., 2003; Schwabe et al., 2005); (2) six annotated genes that to our knowledge were not known to be expressed in surface glia throughout development (*babos*, *Minichromosome maintenance 5*, *Hsp27*, *Major Facilitator Superfamily Transporter 3*, *pericardin*, and *mutagen-sensitive 209*); and (3) six non-annotated genes (*CG10702*, *CG11164*, *CG3168*, *CG4829*, *CG5080*, and *CG6126*). These findings already highlight a good starting point for furthering our knowledge of embryonic BBB formation, maintenance and function. Together with profiling of the embryonic BBB, our adult BBB transcriptome would provide a valuable resource to gain further insight into BBB dynamics during development.

### Neuronal physiology genes identified in the microarray data

Interestingly, we observed some SJ components [e.g., *sinuous* (*sinu*), *scribbled* (*scrib*), *varicose* (*vari*), *Contactin* (*Cont*), and *discs large 1* (*dlg1*)] were enriched in brain and neurons relative to surface glia (Table S2). Assuming these genes are mainly involved in SJ formation, this observation suggests the existence of SJs amongst cells of the adult brain besides the surface glia. Although neuropil glia in the adult CNS ensheath axons (Edwards and Meinertzhagen, 2010), very little has been published on SJs among cells other than the surface glia. It seems likely that other cells form SJs given that this has been found in the peripheral nervous system (Banerjee and Bhat, 2008). Furthermore, enrichment of SJ genes in neurons points to neuron-localized expression of these genes. In the embryonic and larval peripheral nervous system, axons are ensheathed by inner and outer glial membranes involving the expression of SJ proteins Neurexin IV (Nrx-IV), Cont, and Nrg (Banerjee et al., 2006). All three of these proteins are expressed in the glial cells, while Nrg is also expressed by the neurons. Similarly, Nrx-IV is expressed by neurons in the larval CNS where it mediates glial wrapping but is independent of SJ formation (Stork et al., 2008). Given these results, it seems likely that neurons of the adult CNS express SJ components thereby mediating axon insulation by ensheathing glia. This is consistent with axo-glial SJs at the nodes of Ranvier of myelinated axons in the vertebrate nervous system (Bhat et al., 2001; Bhat, 2003).

### BBB genes conserved between drosophila and vertebrates

Using BLAST, we compared the fly and mouse BBB transcriptomes to assess what BBB genes are conserved across evolution. We took a focused approach by targeting known molecular components of the vertebrate BBB covered in reviews by Zlokovic (2008) and Daneman (2012). These proteins are central to the BBB's role as a diffusion barrier (TJs), chemoprotective interface (drug transporters), and conduit for metabolite passage between the blood and brain (SLC transporters). In short, we retrieved sequences for 144 known proteins expressed at the mouse BBB and searched for BLAST homologs (*E* < 10^−5^) in the fly genome. Fly BLAST hits were then annotated with our surface glia transcriptome data to determine which fly homologs are expressed and/or enriched at the BBB.

### Tight and adherens junction proteins

Of the 16 TJ components targeted in our comparative analysis, nine have fly homologs expressed in the surface glia, six of which have homologs enriched in the surface glia (Table 7). According to our methods, the fly genome does not contain true homologs for seven TJ proteins: claudin 3, claudin 5, claudin 12, occludin, immunoglobulin superfamily member 5, peripheral myelin protein 22, and lipolysis stimulated lipoprotein receptor. The importance of some of these proteins to TJ formation is well documented (Hirase et al., 1997; Saitou et al., 2000; Nitta et al., 2003), and the absence of true fly homologs perhaps highlights the differing composition of cell-cell junctions between flies and vertebrates (Wu and Beitel, 2004). We note that claudin-like proteins have been characterized in *Drosophila* (i.e., Sinu, Megatrachea, and Kune-kune), and they are involved in SJ formation (Behr et al., 2003; Wu et al., 2004; Nelson et al., 2010); however, their level of homology with vertebrate claudins is less than the significance level chosen in our BLAST analysis.

**Table 7:** **Fly genes homologous to known junctional components expressed at the mouse BBB**.

**Mouse prot**	**No. exp**.	**Best hit**	**E**	**SG exp**.	**SG/B**	**SG/N**	**No. enr**.	**Fly prot**	**E**	**SG exp**.	**SG/B**	**SG/N**
*Adherens junctions*												
CDH5 (VE-cadherin)	4	CADN	2.E-44	1863		−2.4	0					
CTNNA1 (α-catenin)	3	α-CAT	0	924		−3.7	1	VINC	4.E-13	2475	11.8	29.9
CTNNB1 (β-catenin)	1	ARM	0	3511		−1.9	0					
JUP (γ-catenin)	1	ARM	0	3511		−1.9	0					
PECAM1	1	CG42330	1.E-07	518	−4.9	−1.9	0					
*Tight junctions*												
CASK	37	CASK	0	2260	−2.5	−2.3	5	FOR	2.E-21	697	10.9	77.9
								Unc-89	1.E-29	952	22.5	20.6
								PHKγ	3.E-50	1931	−2.8	2.3
								Lk6	3.E-35	1352	−2.3	2.2
								Par-1	1.E-37	1182	−2.2	1.7
CGNL1 (JACOP)	1	ZIP	6.E-21	2240			0					
ESAM	1	CG42368	4.E-06	271			0					
F11R (JAM-A)	11	ROBO	1.E-10	901	−6.1		4	PPN	7.E-06	3397	35.7	366.7
								CG7981	7.E-06	8162	34.5	37.4
								FAS2	6.E-07	4442	20.3	11.6
								NRG	4.E-07	2645		5.6
MAGI1	15	Magi	4.E-92	297			2	CG33967	3.E-17	997	2.3	6.3
								GRIP	2.E-09	325	10.0	4.5
MARVELD2 (TRIC)	1	Su(Tpl)	4.E-11	1811			0					
MPDZ (MUPP1)	11	Patj	2.E-68	810			1	PYD	2.E-07	3927	5.6	−6.9
TJP1 (ZO1)	3	PYD	6.E-94	3927	5.6	−6.9	1	PYD	6.E-94	3927	5.6	−6.9
TJP2 (ZO2)	4	PYD	2.E-92	3927	5.6	−6.9	1	PYD	2.E-92	3927	5.6	−6.9

The left side of the table lists the number of homologs expressed in the surface glia (SG) followed by the best BLAST hit and its SG expression and enrichments relative to brain (B) and neurons (N). The right side lists all homologs enriched in the surface glia. No significant BLAST hits for Cldn5, Cldn12, Cldn3, Ocln, Igsf5, Pmp22, and Lsr.

The calcium/calmodulin-dependent serine protein kinase (MAGUK family) protein (CASK—same symbol in mouse and fly) is the only highly conserved TJ protein (*E* = 0) co-expressed at both the fly and mouse BBBs. Other strong BLAST hits (*E* < 10^−40^) exist for: membrane associated guanylate kinase, WW and PDZ domain containing 1 (MAGI1); multiple PDZ domain protein (MPDZ); and TJ proteins 1 and 2 (TJP1 and TJP2), which are nearly equally homologous to fly PYD. Interestingly, CASK, MAGI1, TJP1, and TJP2 are all membrane-associated proteins containing guanylate kinase and PDZ domains. MPDZ also contains PDZ domains. These proteins help link transmembrane proteins to the cytoskeleton and bind signaling complexes together (PDZ domain) (Ranganathan and Ross, 1997), and function in signaling themselves (guanylate kinase domain). Our results thus point to strong evolutionary conservation of such proteins at the BBB.

With the exception of PYD, most of the fly homologs of mouse TJ proteins, while expressed in the surface glia, are not specifically enriched. Most of the enriched fly homologs are weakly homologous (*E* > 10^−30^); for example, PPN, FAS2, and CG7981 are highly enriched in the surface glia but are weakly homologous (*E* > 10^−10^) to mouse JAM-A. However, with respect to adherens junction proteins, there are various highly conserved proteins (*E* = 0) co-expressed at the fly and mouse BBBs, which include α-Catenin, and β- and γ-Catenin (equally homologous to fly Armadillo). A high BLAST hit (*E* = 2 × 10^−44^) also exists for VE-cadherin. These results indicate that adherens junction proteins are conserved throughout evolution and function at the BBB in both flies and mice.

Overall, strong conservation between TJ proteins is absent, but we do see conservation between junctional adaptor proteins and adherens junction proteins. These trends were found previously (Knust and Bossinger, 2002) and are not surprising given the ultrastructural differences between the TJ and SJ. In vertebrates, the TJ is apical to the adherens junction, but in *Drosophila* the SJ is basal to the adherens junction. Some of the fly homologs to TJ proteins are localized at a comparable site termed the marginal zone or subapical region (Knust and Bossinger, 2002; Wu and Beitel, 2004), similarly, some of the mouse homologs to SJ proteins are localized at a comparable site termed the basal region (Wu and Beitel, 2004). In conclusion, our results point to the *Drosophila* BBB and SJ complex being a relevant model for the role of adaptor proteins and adherens junction proteins in regulating the diffusion barrier at the BBB.

### Transporters

Besides junctional proteins, the best-studied components of the BBB are the diverse array of ABC and SLC transporters. BLAST hits expressed and enriched at the fly BBB to our targeted set of extensively studied, functionally important mouse BBB transporters (Table 8) reveal striking sequence conservation. For example, mouse BBB transporters that have highly conserved homologs (*E* < 10^−90^) expressed at the fly BBB include: GLUT1, CAT1, LAT1, EAAT1, ABCB1A, BCRP, MRP1, and MRP5. Furthermore, the same homologs for LAT1, ABCB1A, BCRP, MRP1, and MRP5 are not only expressed but also enriched at the fly BBB, and they are among a group of highly conserved homologs enriched at the fly BBB (e.g., there are three fly proteins enriched at the BBB with high homology to ABCB1A). These results point to strong selective pressure for the conservation of BBB-localized transport of glucose, amino acids, and the numerous ABC transporter substrates, thus indicating that across species it is these proteins that are essential for the function of a selective barrier.

**Table 8:** **Fly genes homologous to known transporters expressed at the mouse BBB (for notes on table format, see Table 7)**.

**Mouse prot**	**No. exp**.	**Best hit**	**E**	**SG exp**.	**SG/B**	**SG/N**	**No. enr**.	**Fly prot**	**E**	**SG exp**.	**SG/B**	**SG/N**
Abcb1a (Mdr1a/Pgp)	4	MDR49	0	1485	12.5	33.7	3	Mdr65	0	6078	7.6	298.7
								CG10226	0	3133	7.9	85.5
								MDR49	0	1485	12.5	33.7
Abcc1 (Mrp1)^*^	4	CG5789	0	218	5.7	17.1	4	CG5789	0	218	5.7	17.1
								SUR	2.E-54	111	5.2	4.3
								CG11897	0	2090	5.0	2.8
								MRP	0	2618	3.1	
Abcc5 (Mrp5)^**^	4	MRP	0	2618	3.1		4	CG5789	1.E-166	218	5.7	17.1
								SUR	7.E-52	111	5.2	4.3
								CG11897	1.E-180	2090	5.0	2.8
								MRP	0	2618	3.1	
Abcg2 (Bcrp)	6	W	2.E-76	171	9.9	−2.2	3	CG31689	5.E-52	184	2.3	8.8
								CG3164	1.E-51	610		5.2
								W	2.E-76	171	9.9	−2.2
Atp1a2	8	ATPα	0	15084			2	CG42321	4.E-11	6251	1.5	
								CG31729	2.E-06	1040	2.2	
Lrp1	13	CG8909	0	450	−19.2	−5.4	8	LPR1	1.E-143	3632	15.2	43.2
								CG7981	1.E-44	8162	34.5	37.4
								SLOW	3.E-06	769	12.8	27.1
								SHF	1.E-14	1102	7.6	13.6
								Dl	5.E-11	299	4.7	12.1
								N	1.E-14	362		4.7
								MGL	0	1900	2.0	1.9
								CUE	1.E-23	2734	2.3	
Slc1a1 (EAAT3)^***^	2	EAAT1	2.E-92	2260			0					
Slc1a2 (Eaat2)	2	EAAT2	2.E-90	820	−5.9	−4.9	0					
Slc2a1 (Glut1)	8	GLUT1	1.E-124	711	−3.6	−4.0	6	CG3168	6.E-09	4663	4.2	241.1
								CG4797	2.E-15	5031	32.6	116.1
								CG4607	4.E-25	1021	16.6	59.3
								CG30035	1.E-28	6887	8.1	54.2
								CG6231	2.E-08	1911	23.1	23.8
								ORCT	1.E-05	428	2.2	11.2
Slc4a2	2	CG8177	0	4468			0					
Slc7a1 (Cat1)	5	CG13248	1.E-111	370			2	Jhl-21	6.E-07	1628		9.9
								CG9413	2.E-06	4326	2.1	2.7
Slc7a3 (Cat3)	1	CG13248	1.E-101	370			0					
Slc7a5 (LAT1)	5	JhI-21	1.E-115	1628		9.9	2	Jhl-21	1.E-115	1628		9.9
								CG9413	7.E-82	4326	2.1	2.7
Slc9a1 (Nhe1)	2	NHE1	1.E-45	132	−2.2		0					
Slc16a1 (MCT1)	6	CG3409	1.E-29	1177		−2.4	3	CG8051	4.E-09	1472	117.3	147.8
								KAR	2.E-15	3132	13.7	9.4
								CG13907	2.E-24	4225	3.8	3.9
Slc19a1 (RFC1)	1	CG6574	1.E-51	253	2.6	2.8	1	CG6574	1.E-51	253	2.6	2.8
Slc22a8 (OAT3)	6	ORCT	1.E-60	428	2.2	11.2	6	CG6126	4.E-58	5224	33.1	275.4
								CG3168	5.E-15	4663	4.2	241.1
								CG6231	1.E-26	1911	23.1	23.8
								ORCT2	2.E-57	1166	18.2	12.4
								CG4630	1.E-46	651	8.3	12.2
								ORCT	1.E-60	428	2.2	11.2
Slc38a1 (SNAT1)^****^	1	CG13743	7.E-15	291	−9.5	−8.6	0					
Slc39a10	4	FOI	5.E-63	1643	2.4	1.8	2	CATSUP	1.E-20	430	2.6	2.6
								FOI	5.E-63	1643	2.4	1.8
Slco1c1 (OATP-F)^*****^	1	OATP74D	1.E-63	2041	21.4	64.0	1	Oatp74D	1.E-63	2041	21.4	64.0

Significant BLAST hits that are not expressed in surface glia: Slc19a2, Slc19a3, Slc30a1, and Tfrc.

* Nearly identical results for Abcc4 (Mrp4);

** Nearly identical results for Abcc6 (Mrp6);

*** Nearly identical results for Slc1a3 (Eaat1);

**** Nearly identical results for Slc38a3 (SNAT3) and Slc38a5 (SNAT5);

***** Nearly identical results for Slco1a4 (Oatp2) and Slco2b1 (OATP-B).

Interestingly, we also see that the best BLAST hit is often not the most BBB-enriched homolog. For example, fly GLUT1 is the most homologous fly protein to mouse GLUT1 (*E* = 10^−124^). Fly GLUT1 is expressed at the BBB (surface glia expression = 711), but it is enriched in neurons. However, six other less homologous BLAST hits are highly enriched in surface glia. CG3168 and CG4797 are both annotated as putative sugar transporters with weak homology to GLUT1 (*E* = 6 × 10^−9^ and 2 × 10^−15^, respectively), but they are enriched 214- and 116-fold in surface glia relative to neurons, respectively. We obtained similar results for MCT1 and LRP1, thus indicating that neurons and surface glia in the adult fly brain might use different transporters to transport sugar, monocarboxylates, and lipoproteins, and may allow differential regulation over substrate entry into the brain vs. neuronal uptake.

Overall, we see that the fly and mouse BBBs contain highly homologous transporters, which highlights the importance of these transporters in chemical protection and the transport of metabolites at the BBB regardless of the cell type that expresses them. Our previous investigations on the efflux transporter Mdr65 (Mayer et al., 2009), a fly homolog of ABCB1A, taken together with the elucidation of many more highly homologous BBB transporters in the present study, points to the *Drosophila* surface glia BBB as a relevant model for studying evolutionary conserved BBB-localized transport properties despite the fact that it is of glial rather than endothelial origin.

### Other notable genes co-expressed at the BBB

Noteworthy results among eight additional mouse BBB genes and their fly homologs (Table 9) include the co-enrichment of carbonic anhydrases at the mouse and fly BBBs, which indicates a conserved BBB physiology focused on brain carbon dioxide and bicarbonate homeostasis. We also see very high homology between mouse and fly gamma-glutamyltranspeptidase (GGT1) protein sequences. In vertebrates, GGT1 is expressed at the luminal surface of the BBB where it functions in both amino acid transport and the regulation of glutathione levels (and thus the detoxification processes involving GSTs) (Courtay et al., 1992; Hawkins et al., 2006). An alternative explanation might be that the surface glia express GGT1 similar to astroctyes, where they function to facilitate glutathione synthesis in neurons (Valdovinos-Flores and Gonsebatt, 2012). Another striking result in Table 9 is the conservation of insulin signaling at the mouse and fly BBBs. The *Drosophila* insulin receptor (InR) is the best BLAST hit to both mouse INSR and IGF1R (*E* = 0 for both). Our data suggest that *InR* is expressed in surface glia at low levels; however, another BLAST hit to mouse INSR, CG3837 (*E* = 3 × 10^−75^), is highly enriched in the surface glia. CG3837 was recently identified as a secreted decoy of the insulin receptor (SDR) (Okamoto et al., 2013). SDR acts as an antagonist of insulin signaling and its secretion into the hemolymph by the surface glia of larvae controls body growth; SDR mutants have an abnormally rapid growth rate resulting in larger adult body size. FlyAtlas (Chintapalli et al., 2007) data indicate that *InR* is nearly equally expressed in all tissues, whereas CG3837/*SDR* expression is more restricted, with the highest expression in the CNS. Our microarray data and the data in (Okamoto et al., 2013) suggest that this high CNS expression of CG3837/*SDR* is specifically located in the surface glia (6-fold enriched relative to brain and 154-fold enriched relative to neurons). As *Drosophila* adult body size is predetermined in the larval stages, the maintained expression of CG3837/SDR in the adult surface glia suggests the possibility of novel roles for the adult BBB in insulin regulated physiologies independent of body size control.

**Table 9:** **Fly genes homologous to known genes expressed at the mouse BBB (for notes on table format, see Table 7)**.

**Mouse prot**	**No. exp**.	**Best hit**	**E**	**SG exp**.	**SG/B**	**SG/N**	**No. enr**.	**Fly prot**	**E**	**SG exp**.	**SG/B**	**SG/N**
Bsg	4	ROBO	4.E-10	901	−6.1		1	CG31605	2.E-09	7831	8.1	5.8
Car4	4	CAH2	2.E-26	860	10.0		3	CAH1	8.E-19	799	−2.4	3.6
								CG11284	7.E-18	1756	5.6	2.5
								CAH2	2.E-26	860	10.0	
Ggt1	2	CG4829	1.E-103	1132	−3.6	2.6	1	CG4829	1.E-103	1132	−3.6	2.6
Igf1r	37	INR	0	135			8	CG3837	3.E-78	2097	5.7	153.9
								HTL	2.E-53	422	3.0	19.9
								CG10702	2.E-56	635	3.6	16.5
								FPS85D	2.E-46	1257	14.1	9.7
								PVR	4.E-36	944	5.5	6.1
								PHKγ	1.E-11	1931	−2.8	2.3
								Par-1	1.E-09	1182	−2.2	1.7
								HOP	7.E-40	2470	2.0	−3.3
Insr (IR)	41	INR	0	135			9	CG3837	3.E-75	2097	5.7	153.9
								FOR	3.E-09	697	10.9	77.9
								HTL	1.E-53	422	3.0	19.9
								CG10702	7.E-72	635	3.6	16.5
								Fps85D	7.E-48	1257	14.1	9.7
								PVR	2.E-35	944	5.5	6.1
								PHKγ	9.E-13	1931	−2.8	2.3
								Par-1	1.E-10	1182	−2.2	1.7
								HOP	9.E-37	2470	2.0	−3.3
Kdr (Flk-1)	32	HTL	4.E-68	422	3.0	19.9	7	for	2.E-11	697	10.9	77.9
								UNC-89	2.E-10	952	22.5	20.6
								HTL	4.E-68	422	3.0	19.9
								FPS85D	3.E-31	1257	14.1	9.7
								PVR	1.E-51	944	5.5	6.1
								SGG	5.E-10	6594	1.7	−1.6
Lef1	2	PAN	2.E-51	6202		−1.8	0					
Ptch1	1	PTR	2.E-12	1241	10.1	9.4	1	PTR	2.E-12	1241	10.1	9.4

Lastly, we note that the surface glia also express homologs of the vertebrate BBB proteins LEF1 and PTCH1. LEF1 is a Wnt-responsive transcription factor (Liebner et al., 2008; Stenman et al., 2008; Daneman et al., 2009), and PTCH1 is a mediator of sonic hedgehog signaling (Alvarez et al., 2011). This final set of genes again illustrates that the *Drosophila* surface glia can be used to model evolutionary conserved BBB-localized signaling and regulatory proteins. Overall, our surface glia transcriptome will continue to provide an evolutionary comparative framework as more essential BBB proteins are identified in vertebrates.

## Discussion

Here we have described techniques for the isolation and transcriptome characterization of the adult brain *Drosophila* surface glia. We have shown that our techniques are of high quality yielding a quantitative transcriptomic portrait of the surface glia constituting the BBB. While organ-specific transcriptomes exist for *Drosophila* (Wang et al., 2004; Chintapalli et al., 2007), cell-type-specific data sets have been slower to emerge (Salmand et al., 2011; Berger et al., 2012; Bryantsev and Cripps, 2012; Siddiqui et al., 2012). Ultimately, cell-type-specific transcriptomes will enable us to discern how different cells interact to produce the emergent physiologic properties of a tissue or organ. In our case, we have sampled the two outermost cell layers of the *Drosophila* CNS, and our results confirm that this surface glia layer indeed possesses the hallmarks of a potent chemical protection interface equivalent to the vertebrate brain vascular endothelial BBB component.

That being said, the *Drosophila* model for vertebrate BBB physiology can and should be refined. First, the surface glia transcriptome contains two glial subtypes, the PG and SPG, thus having separate transcriptomes for these cell layers will increase the resolution at which to assign the conserved gene expression patterns discussed in this report. Second, the cortex glia that lie directly underneath the SPG are well positioned to influence BBB properties similar to astrocyte end feet positioning in the vertebrate CNS. Obtaining a transcriptome for these glia will likely add to our understanding of how conserved BBB properties are manifested in the *Drosophila* equivalent of the vertebrate NVU. We have shown that such refinements are feasible by our methodologies, and most importantly, that they are necessary to further research into the mechanisms of development, maintenance, and regulation of conserved BBB properties.

Failure to efficiently circumvent the BBB for the treatment of neurological diseases highlights the complex homeostatic mechanisms that exist at the BBB. Without a model system for which many of the interacting biological processes can be assessed *in vivo*, there will be little progress into understanding BBB development, maintenance, and regulation. The surface glia BBB of *Drosophila* is exactly the model system that is needed. Pharmacokinetics can be measured *in vivo*, hypotheses can be tested with forward and reverse genetics, different cell populations can be manipulated simultaneously, and small molecule modifiers of BBB homeostasis can be found with high-throughput screens. Previously, it was known that the *Drosophila* BBB possesses a few characteristics of the vertebrate BBB; for example, cellular junctions similar to vertebrate TJs (Juang and Carlson, 1994; Schwabe et al., 2005; Stork et al., 2008), a single drug efflux transporter similar to vertebrate ABCB1A (Mayer et al., 2009), and lipoprotein transport (Brankatschk and Eaton, 2010). Now, our characterization of the surface glia transcriptome indicates that numerous processes/structures are evolutionarily conserved between flies and vertebrates. These include: drug efflux (i.e., many B and C class ABC transporters), adherens junctions, insulin signaling, and the basal lamina. The results alone for SLC transporters are staggering. The fly and mouse BBBs co-express highly homologous SLC transporters involved in the transport of amino acids, bicarbonate, organic anions, monocarboxylates, folates, glucose, and zinc. Preliminary deep sequencing of the surface glia transcriptome suggests that about 50% more SLC transporters than revealed by GeneChips are conserved in fly and vertebrate BBBs (data not shown). With this foundation of conserved BBB gene expression patterns, we can begin to perform *in vivo*, translatable experiments in the *Drosophila* BBB model system at a scale unattainable to vertebrate researchers.

### Interesting genes for future study

In addition to various surface glia enriched genes, our investigation revealed two surface glia-specific genes, *Vmat* and *Indy*. Using immunostaining, we showed that DVMAT-B specifically localized to the PG layer of the BBB. DVMAT-B has been shown to localize to the fenestrated glia of the *Drosophila* visual system, where it is thought to function in histamine storage (Romero-Calderon et al., 2008). The fenestrated glia are thought to be the visual system equivalent of PG cells (DeSalvo et al., 2011). Relatively little is known about the contribution of the PG cells to BBB functions; however, the PG-localized expression of DVMAT-B may suggest a role for PG cells in the storage of monoamines, and may function to isolate peripheral and CNS effects of monoamines. *Indy*, the other surface glia-specific gene we identified, encodes a sodium-independent dicarboxylate cotransporter (homologous to mammalian NaDC1, NaDC3, and NaCT) and, like its mammalian counterparts, has been shown to transport intermediates of the Krebs cycle (Rogina et al., 2000; Inoue et al., 2002; Knauf et al., 2002, 2006). Intermediates of the Krebs cycle also have signaling roles, and can act through various G-protein coupled receptors with potential roles in regulating blood pressure and as a hypoxia sensor (Sadagopan et al., 2007; Sapieha et al., 2008). NaDC3 has also been postulated as a glutathione transporter, with implications in oxidative stress regulation (Lash, 2005; Li et al., 2012). NaDC3 and NaCT have been shown to be expressed in neurons and astrocytic glia (Lamp et al., 2011). We have previously shown that *Drosophila* Indy is expressed in both the SPG and the PG layers (DeSalvo et al., 2011), though the polarity of its expression is currently not known. Due to its high similarity to the mammalian SLC13 transporters, its surface glia expression may suggest a role for Indy as a metabolic regulator or sensor of oxidative stress in the BBB. Furthermore, the transcriptional repressor hairy has been suggested as a metabolic switch protein in *Drosophila*, with its upregulation causing hypoxia resistance (Zhou et al., 2008). We found *hairy* to be significantly enriched in the adult surface glia, further suggesting an important metabolic role for the BBB.

We also discovered a leaky BBB phenotype caused by a protein trap in the surface glia-enriched gene *Integrin linked kinase* (*Ilk*). Mammalian integrin linked kinase is involved in transducing signals from the extracellular matrix, through integrins, to initiate downstream intracellular signaling cascades (reviewed in Wu and Dedhar, 2001). Integrin signaling is required to maintain BBB integrity in mammalian endothelial cells; disrupting this critical link between the ECM and intracellular targets led to disruption of TJs and resulted in BBB leakiness (Osada et al., 2011). Our results, therefore, suggest there might be a conserved role in the *Drosophila* BBB for integrin/Ilk signaling from the ECM/basal lamina to regulate BBB integrity. As the role for integrin/Ilk signaling in maintaining BBB integrity has also been linked to collagen composition in the ECM (Gould et al., 2005; Vahedi et al., 2007), it might be of interest to investigate the role of the PG cells, which we have shown to express the collagen IV gene *Vkg*, in neural lamella (basal lamina) composition and SPG septate junction integrity, and the response of PG cells during conditions that perturb BBB function.

Overall, our surface glia transcriptome has identified a number of interesting, conserved genes present in the adult *Drosophila* BBB. We are now well poised to interrogate the interactions between the BBB, underlying neurons and glia, and hemolymph-facing neural lamella to understand the possible feedback mechanisms that occur between the CNS and the whole organism.

### Conflict of interest statement

The authors declare that the research was conducted in the absence of any commercial or financial relationships that could be construed as a potential conflict of interest.
